# Carbon Flow in Acidic CO_2_ Electroreduction

**DOI:** 10.1002/advs.202410679

**Published:** 2025-01-21

**Authors:** Xiao‐Shuang Zhou, Yi‐Yang Bai, Bo Cao, Lin‐Feng Yang, Fu‐Zhi Li, Hai‐Gang Qin, Li‐Na Chen, Long Chen, Jun Gu

**Affiliations:** ^1^ State Key Laboratory of Bio‐Fibers and Eco‐Textiles College of Materials Science and Engineering Collaborative Innovation Center for Marine Biomass Fibers Materials and Textiles of Shandong Province Institute of Marine Biobased Materials Qingdao University Qingdao 266071 China; ^2^ Department of Chemistry Southern University of Science and Technology Shenzhen 518055 China; ^3^ Shenzhen Institute for Advanced Study University of Electronic Science and Technology of China Shenzhen 518110 China

**Keywords:** acidic electrolyte, carbon efficiency, cation exchange membrane, CO_2_ reduction, electrocatalysis

## Abstract

Electrochemical CO_2_ reduction in acidic media attracts extensive research attention due to its potential in increasing carbon efficiency. In most reports, alkali cations are introduced to suppress hydrogen evolution and to promote CO_2_ reduction. However, the mass transport of alkali cations through cation exchange membrane induces the change of electrolyte compositions. Herein, the variation of electrolyte compositions and the flow of carbon during CO_2_ reduction are analyzed quantitatively by simulation and experiments. If the initial amount of alkali cations in the anolyte is higher than the initial amount of H^+^ in the catholyte, the pH of the catholyte increases remarkably in long‐term CO_2_ reduction electrolysis, resulting in the decrease of carbon efficiency. Bicarbonate salt precipitation on the cathode with alkali cation‐containing catholyte is another origin of the decrease of CO_2_ reduction Faradaic efficiency and carbon efficiency. To maintain high carbon efficiency, the electrolyte should contain low concentration of alkali cations or even be free of alkali cations. Decorating the catalyst of cathode with ionomer with high density of cation sites enables CO_2_ reduction in pure acid solution, achieving 30‐h stable carbon efficiency.

## Graphical abstract

The directions of CO_2_ flow in CO_2_ reduction with acidic electrolyte are quantified. When the amount of alkali cation in anolyte is higher than the amount of H^+^ in catholyte, elevation of catholyte pH arises in long‐term electrolysis, causing bicarbonate formation in catholyte and CO_2_ evolution at catholyte/cation exchange membrane interface. Both processes induce a loss of carbon efficiency.

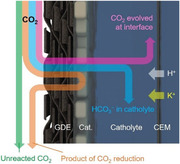

## 1 Introduction

Electrochemical CO_2_ reduction (CO_2_R) at ambient temperature is a developing technique in which renewable electricity is used to convert CO_2_ to valuable fuels and chemicals.^[^ ^1^, ^2^ ^]^ Near neutral electrolyte such as KHCO_3_ solution was first used in this technique.^[^ ^3^ ^]^ Strongly alkaline electrolyte such as concentrated KOH solution was then used in flow cell equipped with gas diffusion electrode (GDE).^[^ ^4^, ^5^ ^]^ Improved selectivity to CO_2_R products and lower overpotential of CO_2_R were achieved with strongly alkaline electrolyte than with near neutral electrolyte. One major issue hinders the application of these techniques is the low carbon efficiency. When strongly alkaline solution is used as the flowing electrolyte, CO_2_ gas is in contact with the alkaline solution. A large fraction of CO_2_ is converted to carbonate (CO_3_^2−^) and the composition of the electrolyte changes over time.^[^ ^6^ ^]^ When near neutral electrolyte is used, the cathodic reaction, CO_2_R or hydrogen evolution reaction (HER), also generates OH^−^ ions which then react with CO_2_ to form CO_3_^2−^ ions. The as‐formed CO_3_^2−^ ions then migrate to the anode and are acidified by H^+^ generated from oxygen evolution reaction (OER) on the anode, releasing CO_2_ with O_2_ at the anode.^[^ ^7^ ^]^ When CO is the product of CO_2_R, the maximum carbon efficiency is 50%. When ethylene or ethanol is the product, the maximum carbon efficiency is 25%.^[^ ^8^ ^]^

**Figure 1:**
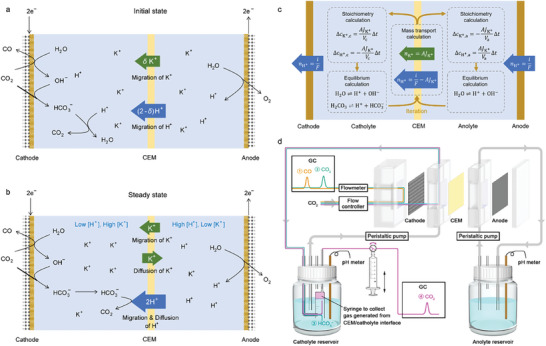
Schematic illustrations of carbon flow in alkali cation‐containing acidic media. a) Initial state. b) Steady state. c) The procedure of the simulation of the evolvement of the electrolyte composition. d) Experimental setup to detect the carbon flow.

To achieve higher carbon efficiency, great efforts were recently devoted to develop CO_2_R techniques with acidic electrolyte.^[^ ^9^, ^10^, ^11^, ^12^ ^]^ As shown in **Figure** **1**a, in acidic electrolyte, the bicarbonate (HCO_3_^−^) species formed from the cathode can be acidified by the hydronium ions (H^+^) in the acidic electrolyte. Then, CO_2_ is released near the cathode and can be reused in the cathodic reaction. Thus, the carbon flow from the cathode to the anode is blocked by the acidic electrolyte.^[^ ^10^ ^]^ The theoretically highest carbon efficiency is 100% if all the inlet CO_2_ is consumed in the cathodic reaction. One major challenge of acidic CO_2_R techniques is to suppress the predominant H^+^ reduction in acidic media. A widely adopted strategy to overcome this challenge is to add alkali cations into the acidic electrolyte.^[^ ^11^, ^13^, ^14^ ^]^ Alkali cations accumulating in the electric double layer of the cathode can shield the electric and thus suppress the migration of H^+^ toward the cathode.^[^ ^13^ ^]^ High cathodic current density can drive a local H^+^ depletion if the migration of H^+^ is effectively inhibited. Besides, alkali cations can stabilize the *CO_2_ intermediate by increasing the electric field strength in the Stern layer of cathode^[^ ^13^ ^]^ and by coordinating directly with the *CO_2_ intermediate.^[^ ^14^, ^15^ ^]^ Both effects promote the kinetics of CO_2_R.

However, since both H^+^ and alkali cations can penetrate the cation exchange membrane (CEM) used to separate the cathode and anode, the composition of the acidic catholyte and anolyte changes over time.^[^ ^16^, ^17^ ^]^ As shown in Figure 1a, the number of H^+^ consumed at the cathode equals the number of H^+^ generated from the anode. Taking K^+^ as an example of alkali cations, due to the mass transport of K^+^ through the CEM, H^+^ gradually accumulates in the anolyte and K^+^ gradually accumulates in the catholyte, resulting in the decrease of anolyte pH and the increase of catholyte pH. As the concentration of K^+^ in the catholyte becomes higher than that in the anolyte, the diffusion of K^+^ from the catholyte to the anolyte can counteract the migration of K^+^ from the anolyte to the catholyte. As shown in Figure 1b, when the diffusion completely cancels the migration of K^+^, the cell reaches the steady state that only H^+^ contributes to the current density through the CEM. If the catholyte becomes neutral in this steady state, the migration of HCO_3_^−^ from the cathode to the CEM is no longer being blocked. HCO_3_^−^ species are then acidified by the H^+^ crossing the CEM and CO_2_ is released at the catholyte‐CEM interface. This part of CO_2_ can hardly be reused in the cathodic reaction. Therefore, when alkali cation‐containing electrolyte was used for acidic CO_2_R, the carbon efficiency may decrease over time.

The migration of alkali cations from the anolyte to the catholyte is the primary cause of pH elevation in the catholyte. Therefore, a potential strategy to prevent this pH increase is to reduce the concentration of alkali cations in the anolyte.^[^ ^10^, ^18^, ^19^ ^]^ In this study, we quantitatively analyzed the carbon flow during acidic CO_2_R through simulations and experiments, correlating the carbon efficiency with the concentration of alkali cations in the acidic electrolyte. Both cases where the catholyte and anolyte had identical or different initial compositions were considered. It was found that when the amount of alkali cations in the anolyte exceeds the amount of H^+^ in the catholyte, significant pH elevation occurs during CO_2_R electrolysis. Another issue induced by alkali cations is the bicarbonate salt precipitation in GDE, resulting in flooding through the GDE. Conducting CO_2_R electrolysis in acidic media with low concentration^[^ ^20^ ^]^ or even the absence of alkali cations^[^ ^21^, ^22^, ^23^ ^]^ is the most efficient way to overcome the as‐mentioned issue. By decorating the cathode catalyst with an ionomer with high density of cationic sites, we achieved stable carbon efficiency in CO_2_R in the acidic electrolyte free of alkali cations.

## 2 Simulations and Experiments

### 2.1 Simulations of the Evolvement of Electrolyte Composition

In general, the membrane region was treated at steady state and the bulk electrolyte was treated at equilibrium state in the simulation. Supplementary Note 1 rationalizes this hypothesis. The flux of H^+^ or K^+^ through the CEM (Nafion 117) from the anolyte to the catholyte can be divided into the diffusive flux and migrative flux. The concentration difference between the anolyte and the catholyte is the driving force for the diffusive flux. The potential difference between the anolyte and catholyte is the driving force for the migrative flux. Therefore, the flux density of H^+^ and K^+^ through the CEM (*J_i_*, *i* = H^+^ or K^+^) can be approximately expressed as:

(1) $$J_{H^{+}}=k_{H^{+},d}\;\left(c_{H^{+},a}-c_{H^{+},c}\right)+k_{H^{+},m}c_{H^{+},a}\left(\varphi_{a}-\varphi_{c}\right)$$

(2) $$J_{K^{+}}=k_{K^{+},d}\;\left(c_{K^{+},a}-c_{K^{+},c}\right)+k_{K^{+},m}c_{K^{+},a}\left(\varphi_{a}-\varphi_{c}\right)$$

The subscript “a” and “c” represent the anolyte and the catholyte, respectively. *c_i_* represents the concentration of species *i* (*i* = H^+^ and K^+^) and *φ* represents the electrolyte potential. *k_i_*_,d_ and *k_i_*_,m_ are the effective rate constants of diffusion and migration of species *i* through the CEM, respectively. Supplementary Note 2 rationalizes Equations (1) and (2). First, the values of *J*_H+_ and *J*_K+_ with different electrolyte compositions and potentials were simulated through Poisson‐Nernst‐Planck (PNP) modeling. The simulation was based on a solution‐membrane‐solution model (Figure S1, Supporting Information), in which the mass transports of H^+^, K^+^ and ClO_4_^−^ (the counter ion) were considered. The simulations were conducted on COMSOL software (v6.1) using the MUMPS solver. Then, the simulated values of *J*_H+_ and *J*_K+_ were used to fit the values of *k_i_*_,d_ and *k_i_*_,m_ (Figure S2, Supporting Information). The detailed procedures of the PNP simulation and the fitting are in the Supporting Information.

According to the PNP simulation, the flux of anions through the CEM is negligible compared with that of cations (Figure S3, Supporting Information). Therefore, we have:

(3) $$J_{K^{+}}+J_{H^{+}}=i/FA$$

In this equation, *i* is the current through the CEM, *F* is the Faraday's constant and *A* is the area of the CEM. By combining Equations ((1), (2), (3)), we can cancel the (*φ*_a_ − *φ*_c_) term. Then, we have:

(4) $$\begin{array}{ccc} & & J_{K^{+}}=[\frac{i}{FA}k_{K^{+},m}c_{K^{+},a}+k_{H^{+},m}k_{K^{+},d}c_{H^{+},a}\left(c_{K^{+},a}-c_{K^{+},c}\right)\\ & & -k_{K^{+},m}k_{H^{+},d}c_{K^{+},a}\left(c_{H^{+},a}-c_{H^{+},c}\right)]\;/\left(k_{H^{+},m}c_{H^{+},a}+k_{K^{+},m}c_{K^{+},a}\right) \end{array}$$

As shown in Figure 1c, the mass transport of K^+^ through the CEM results in a transient change of the concentrations of H^+^ and K^+^ in the catholyte and anolyte in a small interval Δ*t*. The transient change of *c*_H+_ leads to the shift of the equilibria of water autoionization and the dissociation of carbonic acid (H_2_CO_3_). In CO_2_ atmosphere, water autoionization and the second proton dissociation of H_2_CO_3_ were neglected. This approximation is rationalized in Supplementary Note 3. The equilibrium concentrations of H^+^ and K^+^ in the catholyte and the anolyte were then calculated, and the value of *J*_K+_ was calculated again with these renewed concentrations according to Equation (4). By repeating the calculations of the fluxes through the CEM and the equilibrium concentrations (Figure 1c), the evolvement of the compositions of the catholyte and the anolyte was simulated. The detailed procedures of the calculation of equilibrium concentrations and the iteration process are in the Supporting Information.

### 2.2 Preparation of Working Electrode

Cross‐linked poly‐(diallyldimethylammonium chloride) (c‐PDDA) decorated Ag nanoparticles (<100 nm) loaded on the carbon fiber paper (Segracet 29BC) was used as the cathode for CO_2_ reduction to CO. The electrode was prepared with a modified method based on our previous report.^[^ ^22^ ^]^ First, 10 mg of Ag nanoparticles were dispersed in 2 mL of ethanol and sprayed onto the side of micropore layer of 2 pieces of carbon fiber paper (1 × 3 cm^2^) mounted on a heat plate at 120 °C. 100 µL of the aqueous solution of the copolymer of diallyldimethylammonium chloride (DADMACl) and diallylmethylammonia (DAMA) (DADMACl:DAMA ratio = 10:1), 0.5 mL of ethylene glycol, 2.5 mL of ethanol and 30 µL of 1,6‐diiodohexane were mixed and dropped onto 2 pieces of the GDEs with Ag nanoparticles at 150 °C. The electrode was then immersed into the ethanol solution of 1,6‐diiodohexane (4 mL L^−1^) at 72 °C for 12 h. Finally, the electrode was rinsed with ethanol and dried under vacuum.

### 2.3 Measurement of the Carbon Flow

Figure 1d schematically shows the electrolysis cell used to measure the carbon flow. A GDE‐based flow cell was used. IrO_2_ decorated Ti foil was used as the anode. A piece of Nafion 117 was used to separate the catholyte and the anolyte. The effect area of both electrodes and the CEM is 0.5 × 2 cm^2^. The catholyte and the anolyte were circulated by two peristaltic pumps between the cell and two air‐tight bottles as the reservoir for each electrolyte. The volume for each electrolyte was 40 mL. CO_2_ gas was fed into the gas chamber behind the GDE. The inlet flow rate was 2 standard cubic centimeter per minute (sccm), controlled by a mass flow rate controller, and the outlet flow rate was measured by a soap film flowmeter. The probes of pH meters were inserted into the reservoirs of the catholyte and the anolyte to measure the real‐time pH values of the electrolytes. Chronopotentiometry measurements with the current density of −200 mA were conducted at 25 °C on an IVIUM potentiostat (Vertex.20 V.EIS).

As shown in Figure 1d, the CO_2_ molecules flow into the cell have four paths, including: 1. Producing CO molecules; 2. Unreacted CO_2_ molecules through the gas chamber; 3. Dissolved bicarbonate species; 4. CO_2_ molecules evolved from the catholyte‐CEM interface. The gas from the outlet of the gas chamber was analyzed by gas chromatography (GC, GC9790Plus, FULI Instruments). H_2_ was quantified by a thermal conductivity detector (TCD) and CO and unreacted CO_2_ were first converted to methane and then quantified by a fire ionization detector (FID). The concentration of HCO_3_^−^ in the catholyte was quantified by a ThermoFisher ion chromatography (IC, Dionex Aquion RFIC). To conduct the IC measurement, first, 1.00 mL of the catholyte sample for the ionic chromatography analysis was first poured through a Buchner funnel mounted on a flask connected to a recirculating water pump. Then, the solution was purged with Ar for 30 min. Finally, the volume of the sample was fixed to 10.00 mL by adding 50 mm KOH solution. The gas bubbles from the outlet of the catholyte chamber were collected by an upside‐down syringe (Figure S4, Supporting Information) and was analyzed by GC to quantify the CO_2_ evolved from the catholyte‐CEM interface. Figure S5 (Supporting Information) shows the typical GC traces of the gas from the outlet of the gas chamber of the cell (paths 1 and 2) and the collected gas from the outlet of the catholyte (path 4). More experimental details are in the Supporting Information.

## 3 Results and Discussion

**Figure 2:**
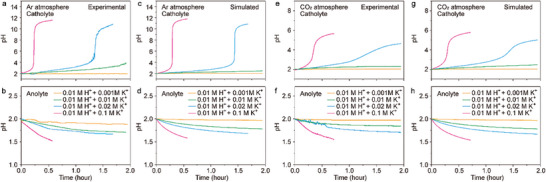
Variation of pH of electrolyte during electrolysis. The experimental (a, b, e and f) and simulated (c, d, g, and h) pH curves of the catholyte (a, c, e and g) and the anolyte (b, d, f and h) under Ar (a‐d) and CO_2_ (e‐h) atmosphere. The initial electrolyte is aqueous solution of 0.01 m of HClO_4_ + 0.001 m KClO_4_ (orange curves), 0.01 m of HClO_4_ + 0.01 m KClO_4_ (green curves), 0.01 m of HClO_4_ + 0.02 m KClO_4_ (blue curves) and 0.01 m of HClO_4_ + 0.1 m KClO_4_ (magenta curves).

We first analyzed the pH variation of the catholyte and anolyte with the identical initial composition. 0.01 M HClO_4_ + *x* M KClO_4_ solution was used as the initial catholyte and anolyte, and the volumes of the catholyte and the anolyte were identical. HClO_4_ is a strong acid in aqueous solution, which simplifies the simulation. The catholyte was saturated by Ar or CO_2_. As shown in **Figure** **2**, the experimental and simulated pH‐time curves are consistent. According to Figure 1c, the mass transport rate of K^+^ from the anolyte to the catholyte equals the consuming rate of H^+^ in the catholyte. When the initial concentration of K^+^ is lower than that of H^+^, the catholyte is still strongly acidic after all K^+^ ions in the anolyte migrate to the catholyte. Therefore, the pH increase of the catholyte is slight, as indicated by the orange curves. When the concentrations of H^+^ and K^+^ are identical, the catholyte pH is slightly higher than that with lower concentration of K^+^, but abrupt pH increase does not occur, as demonstrated by the green curves. In a sharp contrast, when the initial concentration of K^+^ is higher than that of H^+^, an abrupt increase of the catholyte pH can be observed when H^+^ ions in the catholyte are virtually depleted, as shown by the blue and magenta curves. The abrupt pH increase occurs at ca 1.4 h in 0.01 m HClO_4_ + 0.02 m KClO_4_ solution and at ca 0.3 h in 0.01 m HClO_4_ + 0.1 m KClO_4_ solution. Under Ar atmosphere, OH^−^ ions begin to accumulate in the catholyte after the depletion of H^+^, resulting in the final catholyte pH higher than 10 (Figure 2a,c). Under CO_2_ atmosphere, OH^−^ ions generated from the cathode react with CO_2_ to form HCO_3_^−^ ions and the equilibrium between dissolved CO_2_ and HCO_3_^−^ ions results in the final catholyte pH 6 (Figure 2e,g). In contrast to the catholyte, the loss of K^+^ and the accumulation of H^+^ result in pH decrease of the anolyte (Figure 2b,d,f,h). The anolyte with higher initial K^+^ concentration shows more pH decrease.

**Figure 3:**
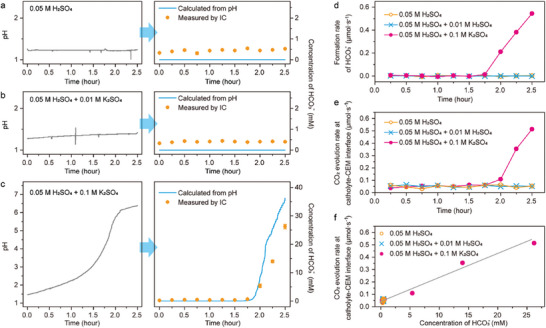
Formation of HCO_3_^−^ and CO_2_ evolution at catholyte‐CEM interface. a–c) pH of the catholyte (left column) and concentration of HCO_3_^−^ in the catholyte (right column) when 0.05 m H_2_SO_4_ a), 0.05 m H_2_SO_4_ + 0.01 m K_2_SO_4_ b) or 0.05 m H_2_SO_4_ + 0.1 m K_2_SO_4_ c) was used as the electrolyte. The blue curves indicate the concentration of HCO_3_^−^ calculated from the pH values and the orange spots indicate the concentration of HCO_3_^−^ measured by IC. The error bars are the standard derivatives of three measurements. d) The formation rate of HCO_3_^−^ in the catholyte determined by ion chromatography. e) The formation rate of CO_2_ at the catholyte‐CEM interface with different electrolyte. f) Dependence of the Formation rate of CO_2_ at the catholyte‐CEM interface on the instant concentration of HCO_3_^−^ in the catholyte.

CO_2_R electrolysis was then conducted with flow cell equipped with GDE (Figure 1d) and the formation of HCO_3_^−^ in the catholyte was detected. Solution of H_2_SO_4_ and K_2_SO_4_ was used as the electrolyte, which is more practical than the combination of HClO_4_ and KClO_4_. When pure H_2_SO_4_ solution was used as the electrolyte, the catholyte pH kept constant over time (**Figure** **3**a). When the initial concentration of K^+^ was lower than that of H^+^, the catholyte pH increased slowly and was still strongly acidic at the end of electrolysis (Figure 3b). When the initial concentration of K^+^ was higher than that of H^+^, the catholyte pH increased significantly and reached ca 6 at the end of electrolysis (Figure 3c). The pH increase of the catholyte would lead to the formation of HCO_3_^−^ from CO_2_ (path 3 in Figure 1d). Since the pH of the catholyte was substantially lower than the p*K*_a2_ of H_2_CO_3_ (10.32), HCO_3_^−^ is the major form of carbon dissolved in the catholyte. Assume the concentration of H_2_CO_3_ in the catholyte obeys Henry's law during CO_2_R electrolysis, the concentration of HCO_3_^−^ in the catholyte can be calculated from the acid‐base equilibrium between H^+^, HCO_3_^−^ and CO_2_:

(5) $$\left[{\mathrm{HCO}}_{3}^{-}\right]=\frac{K_{a1}h_{CO_{2}}p}{\left[H^{+}\right]}\;$$

In this equation, *K*_a1_ is the first dissociation constant of H_2_CO_3_ (4.3 × 10^−7^ M), *h*_CO2_ is the Henry's law constant of CO_2_ (34.5 mol·m^−3^·atm^−1^), *p* is the pressure of CO_2_ gas flow (1 atm). The blue curves in the right column of Figure 3a–c shows the concentration of HCO_3_^−^ calculated from Equation (5). The orange spots in Figure 3a–c shows the concentration of HCO_3_^−^ in the catholyte detected by IC. To conduct the IC analysis, the catholyte was first degassed under vacuum to remove the dissolved CO_2_ and then neutralized by excess KOH to convert HCO_3_^−^ to CO_3_^2−^. The remaining CO_2_ in the catholyte resulted in a background concentration of CO_3_^2−^ ≈ 0.5 mm in the IC analysis, as indicated in Figure 3a–c. After 1.75 h of electrolysis in 0.05 m H_2_SO_4_ + 0.1 m K_2_SO_4_, the concentration of HCO_3_^−^ became significantly higher than the background concentration (Figure 3c). Under this circumstance, the concentration of HCO_3_^−^ measured by IC was lower than that determined from the pH, suggesting that the partial pressure of CO_2_ is considerably lower than 1 atm due to the formation of CO and H_2_ from the cathode. The formation rate of HCO_3_^−^ was then calculated based on the concentration of HCO_3_^−^ measured by IC. As shown in Figure 3d, when 0.05 m H_2_SO_4_ or 0.05 m H_2_SO_4_ + 0.01 m K_2_SO_4_ was used as the electrolyte, the formation rate of HCO_3_^−^ was virtually zero. When 0.05 m H_2_SO_4_ + 0.1 m K_2_SO_4_ was used, the formation rate of HCO_3_^−^ increased drastically after 1.75 h.

As the concentration of HCO_3_^−^ in the catholyte increased, CO_2_ start to evolve from the neutralization reaction between HCO_3_^−^ in the catholyte and H^+^ ions migrate through the CEM. The CO_2_ bubbles were collected from the outlet of catholyte, as illustrated in Figure 1d. Figure 3e shows the formation rate of this part of CO_2_ (path 4 in Figure 1d). During the whole period of electrolysis with 0.05 m H_2_SO_4_ or 0.05 m H_2_SO_4_ + 0.01 m K_2_SO_4_ as the electrolyte, or in the first 1.75 h with 0.05 m H_2_SO_4_ + 0.1 m K_2_SO_4_ as the electrolyte, the pH of the catholyte was lower than 4 (Figure 3a–c). Under this circumstance, the concentration of HCO_3_^−^ in the catholyte is negligible, and the formation rate of CO_2_ in the catholyte was ≈ 0.05 µmol·s^−1^, as shown in Figure 3e. This formation rate of CO_2_ was probably because a small amount of CO_2_ bubbles penetrated the GDE inevitably. The formation rate of CO_2_ at the catholyte‐CEM interface increased drastically after 1.75 h when 0.05 m H_2_SO_4_ + 0.1 m K_2_SO_4_ was used as the electrolyte. The formation rate of this part of CO_2_ was approximately proportional to the instant concentration of HCO_3_^−^ in the catholyte, as shown in Figure 3f.

As revealed by the as‐mentioned experiments, for an acidic CO_2_R electrolysis system with identical volume and initial composition of catholyte and anolyte, the catholyte would become neutral in a long‐term electrolysis if the initial concentration of alkali cations is higher than the concentration of H^+^. In the condition that the volumes of catholyte and anolyte are not identical, the abrupt pH increase would occur if the quantity of alkali cations in the anolyte is higher than the quantity of H^+^ in the catholyte (Figure S6, Supporting Information). The increase of catholyte pH results in the formation of HCO_3_^−^ and further induces evolution of CO_2_ bubbles at the catholyte‐CEM interface, leading to low carbon efficiency. **Table** **1** summarizes the acidic CO_2_R techniques reported in recent years.^[^ ^10^, ^19^, ^21^, ^24^, ^25^, ^26^, ^27^, ^28^, ^29^, ^30^, ^31^, ^32^, ^33^, ^34^ ^]^ In most of these reports acidic electrolyte containing alkali cations was used and the concentration of cations was higher than that of H^+^. SPCE higher than 50% was achieved at the initial stage of the electrolysis in most of these reports, but the SPCE would decrease in long‐term electrolysis due to the formation of HCO_3_^−^ in the catholyte and the evolution of CO_2_ at the catholyte‐CEM interface. A similar issue arises with CEM‐based membrane electrode assembly (MEA) systems for CO_2_R. In MEA setups, the catholyte volume is virtually negligible. As a result, the penetration of alkali cations through the CEM leads to the formation of bicarbonate. Although CO_2_ regenerated at the CEM/cathode interface (path 4) can be reused for CO_2_R, preventing an immediate decrease in carbon efficiency, the accumulation of bicarbonate at the cathode GDE can cause flooding and block the CO_2_ gas diffusion channels. To address this, alkali cation‐free anolyte should be used in CEM‐based MEA systems.

**Table 1:** Summary of reported CO_2_R techniques in flow cell with acidic electrolyte.

Catalyst	Electrolyte	Major product	FE ^a)^	SPCE ^b)^	Reference
Ni‐N‐C	0.005 m H_2_SO_4_ + 1 m Cs_2_SO_4_	CO	100%	76%	[24]
Ni‐SAC/CNTs	0.5 m H_3_PO_4_ + 0.5 m KH_2_PO_4_ +1.5 m KCl	CO	93%	77%	[25]
Bi nanosheets	0.05 m H_2_SO_4_ + 1 m KCl	HCOOH	92%	27%	[26]
SnBi/SiC /PTFE	0.05 m H_2_SO_4_ + 3 m KCl	HCOOH	90%	65%	[19]
Cs_3_Bi_2_Br_9_/C	0.05 m H_2_SO_4_ + 0.5 m CsBr	HCOOH	92%	47%	[27]
Cu_6_Sn_5_	0.05 m H_2_SO_4_ + 3 m KCl	HCOOH	90%	77.4%	[28]
Cu	1 m H_3_PO_4_ + 3 m KCl	C_2+_	48%	77%	[10]
Cu	0.05 m H_2_SO_4_ + 2.5 m KCl	C_2+_	90%	70%	[29]
Cu porous nanosheets	0.05 m H_2_SO_4_ + 3 m KCl	C_2+_	84%	54%	[30]
Pd‐Cu	0.005 m H_2_SO_4_ + 0.5 m K_2_SO_4_	C_2+_	89%	60%	[31]
GDE/Cu/Ni‐N‐C	0.1 m H_2_SO_4_ + 0.4 m K_2_SO_4_	C_2+_	68%	75%	[32]
PTFE/Cu/CoPc	0.5 m H_3_PO_4_ + 0.5 m KH_2_PO_4_ + 2.5 m KCl	C_2+_	76%	90%	[33]
Cu nanoneedles	0.1 m HCl + 3 m KCl	C_2+_	90%	25%	[34]
Cu/cationic groups	0.2 m H_2_SO_4_	C_2+_	80%	90%	[21]
Ag/c‐PDDA	0.05 m H_2_SO_4_	CO	90%	51% ^c^	This work
	0.05 m H_2_SO_4_ + 0.01 m K_2_SO_4_		93%	54%	
	0.05 m H_2_SO_4_ + 0.1 m K_2_SO_4_		96%	58%	

^a)^ The highest FE value in the corresponding report.

^b)^ The highest SPCE value at the initial stage of CO_2_R electrolysis.

^c)^ The SPCE was stable in 30 h.

**Figure 4:**
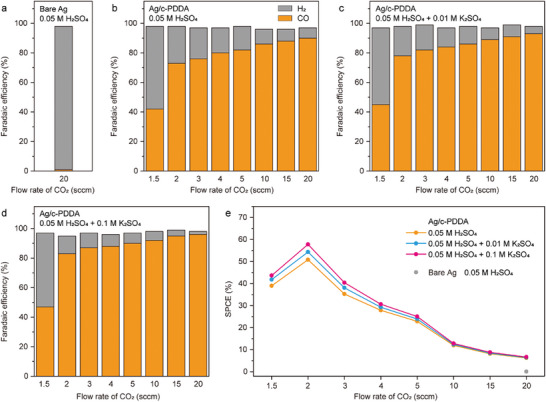
Initial performances in CO_2_R electrolysis. a) FEs of H_2_ and CO of bare Ag nanoparticles in 0.05 m H_2_SO_4_ with the CO_2_ flow rate of 20 sccm. FEs of H_2_ and CO of c‐PDDA decorated Ag nanoparticles in 0.05 M H_2_SO_4_ b), 0.05 m H_2_SO_4_ + 0.01 m K_2_SO_4_ c), and 0.05 m H_2_SO_4_ + 0.1 m K_2_SO_4_ d) with the CO_2_ flow rate varied from 1.5 to 20 sccm. e) SPCE of CO_2_R to produce CO in different electrolytes with varied CO_2_ flow rate.

Alkali cations play essential roles in activate CO_2_ and suppress HER in acidic condition.^[^ ^10^, ^13^, ^14^ ^]^ Quaternary ammonium cations immobilized on the surface of the cathode catalyst may take over the role of alkali cations.^[^ ^22^ ^]^ Herein, we decorated the catalyst of cathode with c‐PDDA as the ionomer with high density of cationic sites. In this cross‐linked polymer, the quaternary ammonium cations are immobilized on the backbones. During CO_2_R electrolysis, the counterions are electrostatically repelled by the cathode, resulting in a positively charged region near the catalyst surface. This region suppresses the migration of H^+^ and increases the interfacial electric field. Ag nanoparticles decorated by c‐PDDA were used as the catalyst. The layer of c‐PDDA covers Ag nanoparticles completely and tightly, as revealed by the transmission electron microscopic images (Figure S7, Supporting Information). The CO_2_R performance of this catalyst was measured in 0.05 m H_2_SO_4_, 0.05 m H_2_SO_4_ + 0.01 m K_2_SO_4_, and 0.05 m H_2_SO_4_ + 0.1 m K_2_SO_4_. The current density was fixed at −200 mA·cm^−2^ and the flow rate of inlet CO_2_ gas was varied. The products were quantified by GC after 15 min of electrolysis. As shown by **Figure** **4**a, bare Ag nanoparticles without c‐PDDA as the ionomer virtually showed no activity toward CO formation from CO_2_R in 0.05 m H_2_SO_4_. As shown by Figure 4b–d, the CO Faradaic efficiency (FE) of c‐PDDA decorated Ag nanoparticles decreased slightly as the flow rate of CO_2_ decreased from 20 sccm to 2 sccm and dropped drastically as the flow rate of CO_2_ decreased to 1.5 sccm. In 0.05 m H_2_SO_4_, the FEs of CO were 90% and 73% at the flow rate of 20 and 2 sccm, respectively (Figure 4b). Figure S8 (Supporting Information) compares the N 1s XPS spectra of the GDE before and after CO_2_R electrolysis in 0.05 m H_2_SO_4_. Both spectra show a peak at 402.0 eV, which is assigned to the quaternary ammonium cations,^[^ ^35^ ^]^ indicating that the c‐PDDA ionomer is stable in acidic media. When K^+^ ions were introduced into the acidic electrolyte, the FE of CO increased slightly. At the flow rate of 20 sccm, the FE of CO in 0.05 m H_2_SO_4_ + 0.01 m K_2_SO_4_ and 0.05 m H_2_SO_4_ + 0.1 m K_2_SO_4_ was 93% and 96% respectively. When the flow rate was decreased to 2 sccm, the FE of CO decreased to 78% and 83%, respectively (Figure 4c,d). Figure 4e further compares the single‐pass carbon efficiency (SPCE) of CO_2_R to produce CO in different electrolytes. For each electrolyte, the maximum SPCE was achieved with the CO_2_ flow rate of 2 sccm. The maximum SPCEs in 0.05 m H_2_SO_4_, 0.05 m H_2_SO_4_ + 0.01 m K_2_SO_4_ and 0.05 m H_2_SO_4_ + 0.1 m K_2_SO_4_ were 51%, 54% and 58%, respectively.

**Figure 5:**
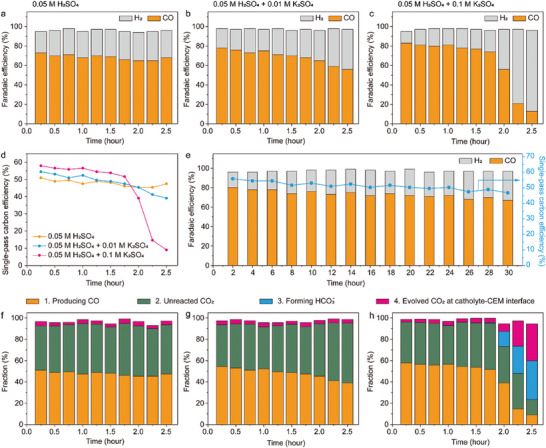
Carbon flow during CO_2_R electrolysis. a–c) FE of CO_2_R on c‐PDDA decorated Ag nanoparticles with 0.05 m H_2_SO_4_ a), 0.05 m H_2_SO_4_ + 0.01 m K_2_SO_4_ b), and 0.05 m H_2_SO_4_ + 0.1 m K_2_SO_4_ c) as the electrolyte. The cathodic current was 200 mA. The flow rate of CO_2_ in the inlet was 2 sccm. d) SPCE of CO formation with different electrolyte. e) FE and SPCE of CO_2_R on c‐PDDA decorated Ag nanoparticles with 0.05 m H_2_SO_4_ as the electrolysis in a 30‐h electrolysis. f–h) Components of carbon flow with 0.05 m H_2_SO_4_ f), 0.05 m H_2_SO_4_ + 0.01 m K_2_SO_4_ g), and 0.05 m H_2_SO_4_ + 0.1 m K_2_SO_4_ h) as the electrolyte. The components include the formation of CO, unreacted CO_2_ in the gas‐phase outlet, the formation of HCO_3_^−^ in the catholyte, and the evolution of CO_2_ bubbles at the catholyte‐CEM interface.

The increase of the FE of CO in the presence of K^+^ ions demonstrate the promotion effect of K^+^ on the kinetics of CO_2_R and the suppression effect of K^+^ on HER. However, the addition of K^+^ ions into the acidic electrolyte would lead to the increase of catholyte pH during electrolysis, resulting in the formation of HCO_3_^−^ in the catholyte and the evolution of CO_2_ at the catholyte‐CEM interface. **Figure** **5**a–c shows the FE of CO_2_R during electrolysis. The inlet CO_2_ flow rate was kept at 2 sccm and the cathodic current density was kept at 200 mA·cm^−2^. With c‐PDDA decorated Ag nanoparticles as the catalyst and 0.05 m H_2_SO_4_ as the electrolyte, the FE of CO kept ≈ 70% during the electrolysis. When 0.05 m H_2_SO_4_ + 0.01 m K_2_SO_4_ was used as the electrolyte, the FE of CO gradually decreased to below 60% at 2.5 h. When 0.05 m H_2_SO_4_ + 0.1 m K_2_SO_4_ was used as the electrolyte, a drastic decrease of FE of CO was observed after 1.75 h, implying that CO_2_ was inadequate under this circumstance due to the formation of HCO_3_^−^. Figure 5d compares the SPCE with different electrolyte during CO_2_R electrolysis. Initially, the SPCE of each electrolyte composition was above 50%. The SPCE with pure acid solution kept ≈ 50%. However, with 0.05 m H_2_SO_4_ + 0.1 m K_2_SO_4_ electrolyte, the SPCE dropped abruptly after 1.75 h and decreased to 9% after 2.5 h. A 30‐h CO_2_R electrolysis was further conducted with c‐PDDA decorated Ag nanoparticles as the catalyst and 0.05 m H_2_SO_4_ as the electrolyte. The FE of CO and the SPCE were stable during the electrolysis (Figure 5e).

The formation of CO is the path 1 and unreacted CO_2_ in the gas‐phase outlet of the flow cell is the path 2 of the carbon flow shown in Figure 1d. Taking these two parts of carbon flow (path 1 and path 2), the formation rate of HCO_3_^−^ in the catholyte (path 3) and the formation rate of CO_2_ bubbles at the catholyte‐CEM interface (path 4) into consideration, we depicted the histograms of the fraction of CO_2_ flow of each path with different electrolytes, as shown in Figure 5f–h. With 0.05 m H_2_SO_4_ or 0.05 m H_2_SO_4_ + 0.01 m K_2_SO_4_ as the electrolyte, the formation of CO (path 1) and unreacted CO_2_ in the gas‐phase outlet of the flow cell (path 2) composed the predominant carbon flow. With 0.05 m H_2_SO_4_ + 0.1 m K_2_SO_4_ as the electrolyte, the formation of HCO_3_^−^ at the cathode (path 3) and CO_2_ evolution at the catholyte‐CEM interface (path 4) became the predominant carbon flow after 1.75 h. Therefore, to maintain high carbon efficiency during long‐term CO_2_R electrolysis, the concentration of alkali cations in the acidic electrolyte needs to be lower than that of H^+^.

**Figure 6:**
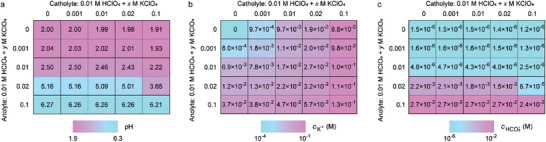
Simulated steady‐state composition of the catholyte with varied initial compositions of the catholyte and the anolyte. a) Steady‐state pH value. b) Steady‐state concentration of K^+^. c) Steady‐state concentration of HCO_3_^−^. The initial catholyte and anolyte contain 0.01 m of HClO_4_ and 0−0.1 m of KClO_4_.

Additionally, we examined the effects of different catholyte and anolyte compositions through numerical simulations and electrochemical measurements. In the simulations, the initial electrolytes consisted of HClO_4_ and KClO_4_, with the HClO4 concentration fixed at 0.01 m and the KClO_4_ concentration varying from 0 to 0.1 m. Figure S9 (Supporting Information) shows the pH value curves of both the catholyte and anolyte during CO_2_R electrolysis, while **Figure** **6** displays the steady‐state pH, K^+^, and HCO_3_^−^ concentrations in the catholyte. For a fixed initial composition in the catholyte, increasing the K^+^ concentration in the anolyte resulted in a higher steady‐state pH of the catholyte (shown along a column in Figure 6a), which in turn led to higher HCO_3_^−^ concentrations in the catholyte (along a column in Figure 6c). However, when the anolyte composition was fixed, increasing the K^+^ concentration in the catholyte slightly lowered the steady‐state pH (along a row in Figure 6a), corresponding to a decrease in HCO_3_^−^ concentration (along a row in Figure 6c). This behavior is attributed to the fact that when the K^+^ concentration in the catholyte exceeds that in the anolyte, the backward diffusion of K^+^ helps mitigate the pH increase in the catholyte. When the K^+^ concentration in the anolyte is lower than the H^+^ concentration in the catholyte (0.01 m in this case), the catholyte remains strongly acidic during CO_2_R electrolysis. Thus, using a catholyte with a high K^+^ concentration and an anolyte with a low K^+^ concentration—especially with a pure acid solution as the anolyte—results in a relatively high K^+^ concentration and low pH in the catholyte at steady state (top right corner of Figure 6b). Under these conditions, CO_2_R is enhanced by the high K^+^ concentration, and the formation of bicarbonate in the catholyte is minimized.

**Figure 7:**
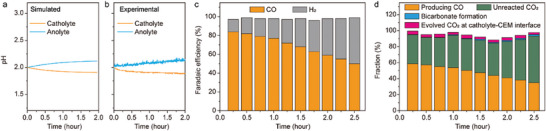
CO_2_R electrolysis with catholyte containing K^+^ and anolyte without K^+^. a) Simulated and b) experimental pH evolution of the catholyte and the anolyte with 0.01 m HClO_4_ + 0.1 m KClO_4_ as the initial catholyte and 0.01 m HClO_4_ as the initial anolyte. c) FE of CO and H_2_ with 0.05 m H_2_SO_4_ + 0.1 m K_2_SO_4_ as the initial catholyte and 0.05 m H_2_SO_4_ as the initial anolyte. d) Components of carbon flow with 0.05 m H_2_SO_4_ + 0.1 m K_2_SO_4_ as the initial catholyte and 0.05 m H_2_SO_4_ as the initial anolyte. The components include the formation of CO, unreacted CO_2_ in the gas‐phase outlet, the formation of bicarbonate precipitate in the GDE, and the evolution of CO_2_ bubbles at the catholyte‐CEM interface.

**Figure** **7**a,b compares the simulated and experimental pH evolutions of the catholyte and anolyte, with 0.01 m HClO_4_ + 0.1 m KClO_4_ as the initial catholyte and 0.01 m HClO_4_ as the initial anolyte. The simulations successfully predicted the trend, showing a slight decrease in the catholyte pH and a slight increase in the anolyte pH over time. Furthermore, CO_2_R electrolysis was performed using a GDE‐based flow cell (Figure 1d) with 0.05 m H_2_SO_4_ + 0.1 m K_2_SO_4_ as the initial catholyte and 0.05 m H_2_SO_4_ as the initial anolyte. The inlet CO_2_ flow rate was 2 sccm, and the cathodic current density was 200 mA·cm^−2^. Figure 7c shows the faradaic efficiencies (FEs) of CO and H_2_ during CO_2_R electrolysis. Compared to the situation where both the catholyte and anolyte were 0.05 m H_2_SO_4_ + 0.1 m K_2_SO_4_ initially (Figure 5c), the FEs of CO were similar until 1.75 h, but after that, the FE of CO was higher when the initial anolyte was only 0.05 m H_2_SO_4_. At 2.5 h, the FE of CO with pure acid as the initial anolyte was 50% (Figure 7c), in contrast to 13% FE of CO when the initial anolyte was 0.05 m H_2_SO_4_ + 0.1 m K_2_SO_4_ (Figure 5c). Similar to Figure 3c,e, during the CO_2_R electrolysis with 0.05 m H_2_SO_4_ + 0.1 m K_2_SO_4_ as the initial catholyte and 0.05 m H_2_SO_4_ as the initial anolyte, the concentration of HCO_3_^−^ in the catholyte was measured by IC, and the formation rate of CO_2_ at the catholyte‐CEM interface was measured by GC, with the results shown in Figure S10 (Supporting Information). Figure 7d illustrates the fraction of CO_2_ flow of each path. At 2.5 h, the fraction of CO formation was 35%, higher than the 9% fraction when the initial catholyte was 0.05 m H_2_SO_4_ + 0.1 m K_2_SO_4_ (Figure 5h). The higher carbon efficiency with the pure acid initial anolyte is attributed to the high acidity of the catholyte, which prevents the formation of HCO_3_^−^, thereby reducing CO_2_ evolution at the catholyte‐CEM interface.

At 2.5 h, the fraction of CO formation was 35% with 0.05 m H_2_SO_4_ + 0.1 m K_2_SO_4_ as the initial catholyte and 0.05 m H_2_SO_4_ as the initial anolyte (Figure 7d), which was still lower than the 47% observed with 0.05 m H_2_SO_4_ as both the initial catholyte and anolyte (Figure 5f). This difference was due to bicarbonate precipitation in the cathode GDE when the catholyte contained K^+^. To quantify bicarbonate formation, the cathode GDE was rinsed with deionized water from the gas‐chamber side every 0.25 h, and the quantity of HCO_3_^−^ was measured by IC, as shown in Figure S11 (Supporting Information). The “bicarbonate formation” columns in Figure 7d represent the fraction of CO_2_ converted into bicarbonate precipitate. Bicarbonate precipitation impaired the GDE's hydrophobicity and caused flooding, which in turn reduced the FE of CO and overall carbon efficiency. Figure S12 (Supporting Information) shows the energy dispersive spectroscopy (EDS) mapping of the cross‐section of the working electrode after CO_2_R electrolysis in the acidic electrolytes containing K_2_SO_4_. With 0.05 m H_2_SO_4_ + 0.01 m K_2_SO_4_ as the electrolyte, the aggregation of K element can be observed (Figure S12a, Supporting Information), indicating the salt precipitation in the GDE. With 0.05 m H_2_SO_4_ + 0.1 m K_2_SO_4_ as the electrolyte, larger amount of salt precipitation was observed (Figure S12b, Supporting Information). Figure S13 (Supporting Information) compares the C 1s and K 2p XPS spectra of the GDE after CO_2_R electrolysis in 0.05 m H_2_SO_4_ + 0.1 m K_2_SO_4_ and in 0.05 m H_2_SO_4_. When K^+^‐containing acidic electrolyte was used, the C 1s peak of HCO_3_^−^ and K 2p^3/2^ and K 2p^1/2^ peaks were seen. When K^+^‐free acidic electrolyte was used, the C 1s peak of HCO_3_^−^ was not observed, indicating no bicarbonate precipitate formed. Therefore, conducting CO_2_R electrolysis in acidic electrolyte free of alkali cations is the most effective strategy to keep the high carbon efficiency. In this study, CO_2_R in alkali cation‐free acidic media was achieved by decorating the catalyst with an ionomer containing a high density of cationic sites. This design creates a localized high‐pH region and enhances the electric field at the catalyst‐catholyte interface, effectively suppressing H^+^ reduction and promoting CO_2_R. Additionally, covering the catalyst with a hydrophobic organic adlayer can facilitates CO_2_R by limiting H^+^ diffusion and increasing the local concentration of CO_2_.^[^ ^20^ ^]^ Developing catalysts with intrinsically high activity for CO_2_R and low activity for hydrogen evolution under acidic conditions warrants further research and exploration. Cobalt phthalocyanine supported on carbon nanotubes is an example for CO_2_R to produce CO in pure acid electrolyte.^[^ ^23^ ^]^

In this study, CO formation on an Ag catalyst was used as a model CO_2_R process in acidic media. When formic acid is the product of CO_2_R, its buffer capacity can help mitigate pH elevation in the catholyte caused by alkali cation penetration through the CEM. Since the p*K*_a_ of formic acid (3.7) is lower than the p*K*_a1_ of H_2_CO_3_ (6.4), H^+^ consumption in the catholyte initially leads to the conversion of formic acid to formate. However, if the concentration of alkali cations in the anolyte is sufficiently high, significant HCO_3_^−^ formation in the catholyte can occur once most of the formic acid has been converted to formate, leading to the loss of carbon efficiency.

A CEM is typically used to separate the catholyte and anolyte during CO_2_R in acidic media. Replacing the CEM with an anion exchange membrane (AEM) results in H^+^ ions being consumed by the cathodic reaction, while anions in the catholyte migrate through the AEM to the anolyte. This process depletes the acid electrolyte in the catholyte. If the initial catholyte contains only acid, the depletion causes a significant increase in catholyte resistance, eventually rendering it incapable of sustaining the current density (Figure S14a, Supporting Information). If the initial catholyte also contains alkali salts, bicarbonate salts accumulate once the acid is depleted, ultimately converting the catholyte into a bicarbonate solution (Figure S14b, Supporting Information).

A potential strategy to maintain low pH and a high concentration of alkali cations in the catholyte is to use a bipolar membrane (BPM) in reverse mode instead of a CEM (Figure S14c, Supporting Information). In this configuration, the BPM's anion exchange layer blocks the migration of alkali cations between the anolyte and catholyte. An alkaline solution can be used as the anolyte, while an acidic solution containing alkali cations can serve as the catholyte. The primary challenge of a BPM‐based electrolysis system, however, lies in the sluggish kinetics of water dissociation at the interface between the cation exchange layer and the anion exchange layer of the BPM.^[^ ^36^ ^]^

## 4 Conclusion

Techniques of CO_2_R in acidic conditions are developed to improve the carbon efficiency. Alkali cations are introduced into the acidic electrolyte to promote CO_2_R and suppress HER. This work theoretically and experimentally demonstrated that if the initial amount of alkali cations in the anolyte is higher than the amount of H^+^ in the catholyte, the migration of alkali cations through the CEM would lead to abrupt elevation of catholyte pH in long‐term CO_2_R electrolysis. The pH elevation induces the formation of HCO_3_^−^ in the catholyte and the evolution of CO_2_ bubbles at the catholyte‐CEM interface. These two processes result in the loss of carbon efficiency. Bicarbonate precipitation in the cathode GDE with alkali cation‐containing catholyte is another issue causing the decrease of CO_2_R Faradaic efficiency and carbon efficiency. Decorating the catalyst with c‐PDDA as the ionomer enabled CO_2_R in acidic electrolyte free of alkali cations, improving the stability of carbon efficiency during CO_2_R electrolysis.

## Conflict of Interest

The authors declare no conflict of interest.

## Supporting information



Supporting Information

## Data Availability

The data that support the findings of this study are available from the corresponding author upon reasonable request.
