# Supplementary material for: Carbon Flow in Acidic CO2 Electroreduction
Source: Adv Sci (Weinh). 2025 Jan 21;12(10):2410679. doi: 10.1002/advs.202410679 (PMC11905065; doi:10.1002/advs.202410679)
Supplement: Supplementary file 1 — Supporting Information [file ADVS-12-2410679-s001.pdf]

ADVANCED SCIENCE
Open Access

Supporting Information

for *Adv. Sci.*, DOI 10.1002/advs.202410679

Carbon Flow in Acidic CO_2 Electroreduction

Xiao-Shuang Zhou, Yi-Yang Bai, Bo Cao, Lin-Feng Yang, Fu-Zhi Li, Hai-Gang Qin, Li-Na Chen, Long Chen* and Jun Gu**

Supporting Information of

Carbon Flow in Acidic CO_2 Electroreduction

AUTHORS: Xiao-Shuang Zhou[1,2], Yi-Yang Bai[2], Bo Cao[2], Lin-Feng Yang[2], Fu-Zhi Li[2], Hai-Gang Qin[2], Li-Na Chen[3]*, Long Chen[1]*, Jun Gu[2]*

AFFILIATION:

1. State Key Laboratory of Bio-Fibers and Eco-Textiles, College of Materials Science and Engineering, Collaborative Innovation Center for Marine Biomass Fibers, Materials and Textiles of Shandong Province, Institute of Marine Biobased Materials, Qingdao University, Qingdao, 266071, China

2. Department of Chemistry, Southern University of Science and Technology, Shenzhen, 518055, China.

3. Shenzhen Institute for Advanced Study, University of Electronic Science and Technology of China, Shenzhen, 518110, China.

Corresponding author E-mail: linachen@hit.edu.cn; chenlong@qdu.edu.cn; guj6@sustech.edu.cn

SIMULATION SECTION

PNP simulations:

Poisson-Nernst-Planck modeling was used to simulate the mass transport process through the CEM. Figure S1 shows the geometry, governing equations and the boundary conditions used in the simulations. A Nafion 117 membrane with the thickness of 183 μm was used as the membrane.[1] A 100 μm-thick diffusion layer was added on each side of the membrane. The density of the fixed charge in the membrane (ρ_{fix}) was −96 C·cm^{-3}.[2] Mixed solution of $HClO_4$ and $KClO_4$ was used as both the catholyte and the anolyte. The mass transports of H^+, K^+ and ClO_4^- were considered in the simulations.

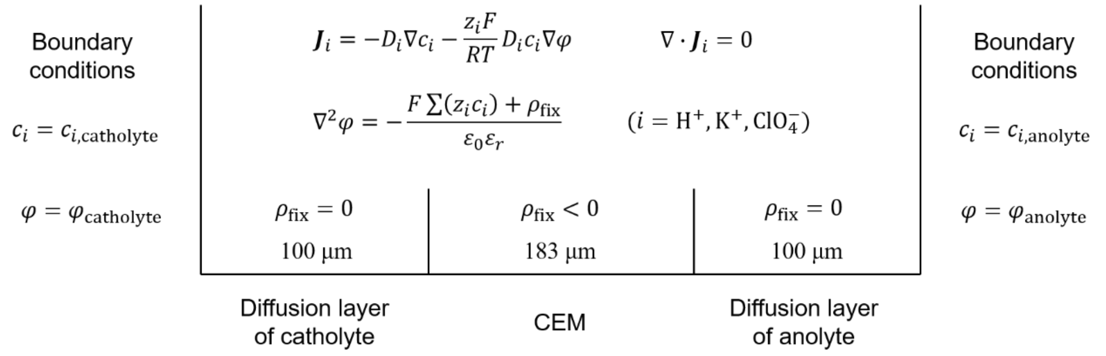

Figure S1. Models for the simulation of mass transport through the CEM.

During the electrolysis, the catholyte and the anolyte flow through the two chambers separated by them membrane. The forced convection makes the bulk parts of the catholyte and the anolyte in uniform and equilibrium states. The electrolyte close to the membrane does not flow. The effective thickness of the diffusion layer (δ_d) on a solid surface within laminar flow can be estimated according to:

$$\delta_{\mathrm{d}} \approx D^{1/3} v^{1/6} x^{1/2} v_0^{-1/2} \quad \text{(S1)}$$

In this equation, D is the diffusion coefficient, v is the kinematic viscosity, x is the length of the solid surface and v_0 is the bulk velocity of the flow. When taking the diffusion coefficient of H^+ (9.31×10^{-9} m^2·s^{-1}), kinematic viscosity of water at 298 K (1×10^{-6} m^2·s^{-1}), effective length of the membrane (2×10^{-2} m) and the velocity of the electrolyte flow (0.1 m·s^{-1}) into this equation, we get $\delta_d \approx$ 94 μm. Therefore, it is reasonable to set

the thickness of the diffusion layer at 100 μm. In summary, the bulk electrolyte is uniform and at equilibrium state due to forced convection, and the diffusion layers with the thickness around 100 μm exist at the membrane-electrolyte interfaces.

Nernst-Planck equation was used to describe the mass transport of the ionic species:

$$\boldsymbol{J}_i = -D_i \nabla c_i - \frac{z_i F}{RT} D_i c_i \nabla \varphi \qquad \text{(S2)}$$

In this equation, $\boldsymbol{J}_i$, D_i, c_i and z_i are the flux density, diffusion coefficient, concentration and number of charge of species i, respectively. F is the Faraday's constant, R is the gas constant, T is the temperature and φ is the potential, respectively. Compared with the quantity of ions in the bulk electrolyte, the quantity of ions in this membrane region is negligible. Therefore, this membrane region can reach a steady state before any significant change of the bulk electrolyte. Thus, the model was solved under steady state:

$$\nabla \cdot \boldsymbol{J}_i = 0 \qquad \text{(S3)}$$

Poisson equation was used to correlate the potential and the spatial charge density:

$$\nabla^2 \varphi = -\frac{F \sum(z_i c_i) + \rho_{\mathrm{fix}}}{\varepsilon_0 \varepsilon_r} \qquad \text{(S4)}$$

In this equation, ρ_{fix} is the density of the fixed charge ($\rho_{\mathrm{fix}} = -96\ \mathrm{C\cdot cm^{-2}}$ in the membrane and $\rho_{\mathrm{fix}} = 0$ in the diffusion layers), ε_0 is the permittivity of vacuum and ε_r is the relative permittivity. Dirichlet boundary conditions were used for the concentrations and potential at both sides. The values of parameters used in the simulation are listed in Table S1.

Fitting of effective diffusive and migrative rate constants:

The fluxes of species i ($i = H^+$ or K^+) through the CEM is expressed as:

$$J_i = k_{\mathrm{d},i}(c_{i,\mathrm{a}} - c_{i,\mathrm{c}}) + k_{\mathrm{m},i} c_{i,\mathrm{a}} (\varphi_{\mathrm{a}} - \varphi_{\mathrm{c}}) \qquad \text{(S5)}$$

In this equation, the subscript 'a' and 'c' represent the anolyte and the catholyte, respectively. c_i represent the bulk concentration of species i in the electrolyte. φ

represents the potential of the bulk electrolyte. $k_{d,i}$ and $k_{m,i}$ are defined the effective rate constants of diffusion and migration of species i through the CEM, respectively. With given electrolyte composition ($c_{H+,a}$, $c_{H+,c}$, $c_{K+,a}$ and $c_{K+,c}$) and potentials (φ_a and φ_c), the values of J_{H+} and J_{K+} were obtained from the as-mentioned PNP simulation. Then, the values of $k_{d,H+}$, $k_{m,H+}$, $k_{d,K+}$ and $k_{m,K+}$ were obtained by fitting the values of J_{H+} and J_{K+} at different conditions.

Specifically, to fit the value of $k_{d,H+}$, $c_{H+,c}$, $c_{K+,a}$ and $c_{K+,c}$ were fixed at 0.01 M, φ_a and φ_c were fixed at 0 V, and $c_{H+,a}$ was varied. Then, $k_{d,H+}$ is the slope of J_{H+}- $c_{H+,a}$ plot (Figure S2a). To fit the value of $k_{d,K+}$, $c_{H+,a}$, $c_{H+,c}$, and $c_{K+,c}$ were fixed at 0.01 M, φ_a and φ_c were fixed at 0 V, and $c_{K+,a}$ was varied. Then, $k_{d,K+}$ is the slope of J_{K+}- $c_{K+,a}$ plot (Figure S2b). To fit the value of $k_{m,H+}$ and $k_{m,K+}$, $c_{H+,a}$, $c_{H+,c}$, $c_{K+,a}$ and $c_{K+,c}$ were fixed at 0.1 M, φ_c was fixed at 0 V and φ_a was varied. Then, the slope of J_{H+}- φ_a plot is $k_{m,H+}\cdot c_{H+,a}$ (Figure S2c), and the slope of J_{K+}- φ_a plot is $k_{m,K+}\cdot c_{K+,a}$ (Figure S2d).

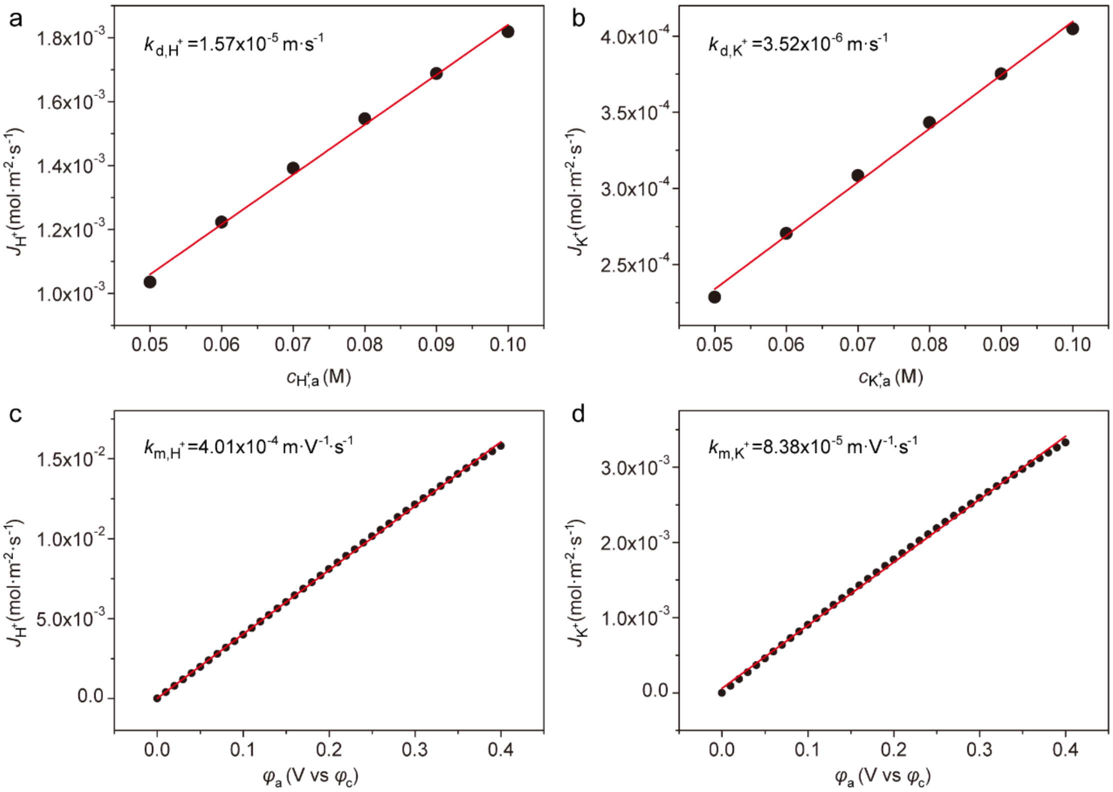

Figure S2. Fitting of effective diffusive and migrative rate constants. (a) J_{H+}-$c_{H+,a}$ plot. (b) J_{K+}-$c_{K+,a}$ plot. (c) J_{H+}-φ_a plot. (d) J_{K+}-φ_a plot.

Equilibrium calculation:

According to Figure 1c of the main text, the transient change of the concentration of H^+ in the anolyte and the catholyte after the small interval (denoted as Δ_a and Δ_c, respectively) can be expressed as:

$$\Delta_a = \frac{AJ_{K^+}}{V_a}\Delta t \quad (S6)$$

$$\Delta_c = -\frac{AJ_{K^+}}{V_c}\Delta t \quad (S7)$$

In this equation, V_c and V_a represent the volume of the catholyte and the anolyte, respectively. When the electrolyte does not contain any species with buffer capacity, water autoionization is the only equilibrium need to be considered. This situation applies to the catholyte fed by Ar and the anolyte. Then, an equilibrium chat is listed as below:

	$H_2O \rightleftharpoons$	H^+	OH^-
Initial		c_{H^+}	K_w/c_{H^+}
After transient mass transport of H^+		$c_{H^+} + \Delta$	K_w/c_{H^+}
Equilibrium		$c_{H^+} + \Delta - x$	$K_w/c_{H^+} - x$

In the chat above, K_w is the equilibrium constant of water autoionization. Then, we have:

$$(c_{H^+} + \Delta - x) \cdot (K_w/c_{H^+} - x) = K_w \quad (S8)$$

Therefore,

$$x = \frac{1}{2}\left[K_w/c_{H^+} + c_{H^+} + \Delta - \sqrt{(K_w/c_{H^+} + c_{H^+} + \Delta)^2 - 4\,K_w\Delta/c_{H^+}}\right] \quad (S9)$$

When the electrolyte contains weak acid and the conjugate base, such as the catholyte with dissolved CO_2, the dissociation equilibrium of the weak acid (H_2CO_3) determines the equilibrium concentration of H^+. We assume the catholyte keeps saturated by CO_2. Then, an equilibrium chat is listed as below:

	H_2CO_3 ⇌	H^+	HCO_3^-
Initial	hp_{CO_2}	c_{H^+}	$K_{a1}hp_{CO_2}/c_{H^+}$
After transient mass transport of H^+	hp_{CO_2}	$c_{H^+} + \Delta$	$K_{a1}hp_{CO_2}/c_{H^+}$
Equilibrium	hp_{CO_2}	$c_{H^+} + \Delta - x$	$K_{a1}hp_{CO_2}/c_{H^+} - x$

In the chat above, h is the Henry's law constant of CO_2, p_{CO2} is the partial pressure of CO_2 in the gas fed to the catholyte. In our simulation, we set p_{CO2} to 1 atm. K_{a1} is the first dissociation constant of H_2CO_3. Then, we have:

$$(c_{H^+} + \Delta - x) \cdot (K_{a1}hp_{CO_2}/c_{H^+} - x)/(hp_{CO_2}) = K_{a1} \quad \text{(S10)}$$

Therefore,

$$x = \frac{1}{2}[K_{a1}hp_{CO_2}/c_{H^+} + c_{H^+} + \Delta - \sqrt{(K_{a1}hp_{CO_2}/c_{H^+} + c_{H^+} + \Delta)^2 - 4K_{a1}hp_{CO_2}\Delta/c_{H^+}}] \quad \text{(S11)}$$

The equilibrium concentration of H^+ in the anolyte and the catholyte after a time interval (Δt) during which H^+ ions transport through the CEM can be expressed as:

$$c_{H^+,a}(t + \Delta t) = c_{H^+,a}(t) + \Delta_a - x_a \quad \text{(S12)}$$

$$c_{H^+,c}(t + \Delta t) = c_{H^+,c}(t) + \Delta_c - x_c \quad \text{(S13)}$$

Since K^+ ions do not participate in any solution-phase reactions, the concentration of K^+ after the time interval can be expressed as:

$$c_{K^+,a}(t + \Delta t) = c_{K^+,a}(t) - \Delta_a \quad \text{(S14)}$$

$$c_{K^+,c}(t + \Delta t) = c_{K^+,c}(t) - \Delta_c \quad \text{(S15)}$$

Simulation of the pH evolution of the electrolyte:

The pH evolutions of the anolyte and the catholyte during electrolysis with constant current density were simulated. $\Delta t = 2$ s was used as the time interval. First, based on the concentrations of H^+ and K^+ in the anolyte and the catholyte, the value of J_{K+} was calculated according to Equation 4 in the main text. Second, the concentrations of H^+ and K^+ in the anolyte and the catholyte after the time interval were calculated according to Equation S9-S12. These two steps were repeated to calculate the compositions of the catholyte and the anolyte after certain number of iterations.

EXPERIMENTAL SECTION

Preparation of the copolymer:

First, 6.9 mL of the aqueous solution of diallyldimethylammonium chloride (DADMACl, 60 wt.%, Macklin), 1.1 mL of diallylmethylamine (DAMA, 99.5%, Bide) and 0.20 mL of condensed H_2SO_4 (98%, ultrapure for trace metal analysis, Aladdin) were mixed to form a uniform solution. Next, 20 mg of $(NH_4)_2S_2O_8$ (99.99%, Macklin) and 20 mg of ethylenediaminetetraacetic acid disodium salt (Na_2EDTA, 98%, Bide) were added into this solution. This solution was stirred at 60 °C in Ar atmosphere for 4 hours. White precipitate was then formed by adding 10 mL of acetone. The precipitate was washed by ethanol for 3 times to give the copolymer of DADMAC and DAMA.

Electrochemical measurements:

H_2SO_4 (98%, trace metal basis), K_2SO_4 (99.995%), $HClO_4$ (70~72%, purity 99.999%) and KOH (99.999%) were used to prepare the electrolyte. H_2 and CO were the only products of the cathodic reaction. The FE of CO was calculated according to:

$$\mathrm{FE_{CO}} = \frac{2X_{\mathrm{co}}pv_{\mathrm{out}}/RT}{I/F} \tag{S16}$$

In this equation, X_{CO} is the volume fraction of CO in the outlet gas obtained from GC analysis, p is the ambient pressure (101.3 kPa), v_{out} is the outlet gas flow rate measured by the soap film flowmeter, R is the gas constant (8.314 $J \cdot K^{-1} \cdot mol^{-1}$), T is the temperature (298 K), I is the current of chronopotentiometry experiment (200 mA), F is the Faraday's constant (96485 $C \cdot mol^{-1}$). The SPCE of CO was calculated according to:

$$\mathrm{SPCE} = \frac{I \cdot \mathrm{FE_{co}}/2}{Fpv_{\mathrm{in}}/RT} \tag{S17}$$

In this equation, m_i is the number of carbon atom in one molecule i, and v_{in} is the inlet flow rate of CO_2.

The fraction of CO formation (path 1) among the total amount of CO_2 in the inlet of the cell equals the SPCE. The fraction of unreacted CO_2 fraction in the outlet of the gas chamber (path 2) is calculated according to:

$$\text{Fraction 2} = \frac{X_{CO_2} v_{out}}{v_{in}} \quad \text{(S18)}$$

In this equation, X_{CO2} is the volume fraction of CO_2 in the outlet gas obtained from GC analysis.

The fraction of the formation of HCO_3^- (path 3) is calculated according to:

$$\text{Fraction 3} = \frac{[c_{HCO3-}(t) - c_{HCO3-}(t-\Delta t)]V_{catholyte}}{\Delta t \cdot p v_{in}/RT} \quad \text{(S19)}$$

In this equation, $c_{HCO3-}(t)$ is the concentration of HCO_3^- in the catholyte determined by ion chromatography (IC) at the time t, Δt is the time interval for IC sampling and $V_{catholyte}$ is the volume of the catholyte.

The fraction of CO_2 evolved at the catholyte-CEM interface (path 4) is calculated according to:

$$\text{Fraction 4} = \frac{V_{bubbles} X_{CO_2}}{\Delta t \cdot v_{in}} \quad \text{(S20)}$$

In this equation, $V_{bubbles}$ is the volume of gas collected from the outlet of the catholyte chamber by the syringe in a time interval Δt and XCO_2 is the volume fraction of CO_2 in the gas collected in the syringe determined by GC.

Supplementary Note 1. Hypothesis about the membrane region and bulk electrolyte in the simulations

Since the volume of the bulk electrolyte (dozens of cm^3) is much larger than the volume of the membrane region (membrane and the diffusion layers, ~0.04 cm^3), the composition change of the bulk electrolyte caused by the flux through the membrane can be regarded as infinitesimal. The composition change of the bulk electrolyte is quite slow at hour-scale. Considering the rate constant for the neutralization reaction between OH^- and H^+ or CO_2:

$OH^- + H^+ \rightarrow H_2O$ ($k_1 = 2.4\times10^9$ $M^{-1}\cdot s^{-1}$) (S21)

$OH^- + CO_2 \rightarrow HCO_3^-$ ($k_2 = 6.0\times10^9$ $M^{-1}\cdot s^{-1}$) (S22)

Since the concentration of OH^- is much lower than that of H^+ or CO_2 in the electrolyte, the half-time of the above reactions can be estimated according to:

$$t_{1/2} = \frac{\ln 2}{k_1[\mathrm{H^+}]} = 2.9 \times 10^{-8}\ \mathrm{s} \quad \text{(S23)}$$

or

$$t_{1/2} = \frac{\ln 2}{k_2[\mathrm{CO_2}]} = 3.5 \times 10^{-9}\ \mathrm{s} \quad \text{(S24)}$$

The half-times of the homogeneous reactions are much shorter than the time for the composition change of the bulk electrolyte. Therefore, it is reasonable to treat the bulk electrolyte at uniform and equilibrium state.

Moreover, for the membrane, the time to reach steady state can be estimated according to:

$$t = \frac{d^2}{\pi D} \quad \text{(S25)}$$

Taking the thickness of Nafion 117 membrane (183 μm) and the diffusion coefficient of H^+ in the membrane (4.6×10^{-10} $m^2\cdot s^{-1}$) into the calculation, the time to reach steady state is 23 s, also much shorter than the time for the composition change of the bulk electrolyte. Therefore, it is reasonable to treat the membrane region at steady state.

Supplementary Note 2. Effective rate constants of diffusion and migration

We divided the flux of a cationic species (H^+ or K^+) through the membrane into two parts, namely diffusion and migration. The concentration difference between the two sides of the membrane is the driving force of diffusion. Therefore, we defined $k_{i,\mathrm{d}}$ (i = H^+ or K^+) as the ratio between the diffusion rate and the concentration difference. The potential difference between the two sides of the membrane is the driving force of migration. Therefore, we defined $k_{i,\mathrm{m}}$ (i = H^+ or K^+) as the ratio between the migration rate and the potential difference and the concentration of species i.

The local diffusive flux is linear to the local concentration gradient and the local migrative flux is linear to the local potential gradient and the local concentration. Considering that the concentration of species i varies at different position in the membrane, the local diffusive and migrative fluxes of species i also vary at different position in the membrane. However, the steady-state total flux can be expressed as the sum of two parts, respectively linear to the concentration difference between the two sides and linear to the potential difference between the two sides and the concentration of one side, as demonstrated in following.

The local total flux density of species i in the membrane expressed as:

$$J_i = -D_i \frac{\mathrm{d}c_i}{\mathrm{d}x} - \frac{F}{RT} D_i c_i \frac{d\varphi}{dx} \tag{S26}$$

In this equation, D_i is the diffusion coefficient, c_i is the concentration, F is the Faraday constant, R is the gas constant, T is the temperature, φ is the potential. The membrane-anolyte interface is defined as $x = 0$ and the membrane-catholyte interface is defined as $x = d$. d is the thickness of the membrane. At steady state,

$$\frac{\mathrm{d}c_i}{\mathrm{d}t} = -\frac{\mathrm{d}J_i}{\mathrm{d}x} = D_i \frac{\mathrm{d}^2 c_i}{\mathrm{d}x^2} + \frac{FD_i}{RT}\frac{\mathrm{d}c_i}{\mathrm{d}x}\frac{\mathrm{d}\varphi}{\mathrm{d}x} + \frac{F}{RT} D_i c_i \frac{\mathrm{d}^2\varphi}{\mathrm{d}x^2} = 0 \tag{S27}$$

Assuming the electroneutrality within the membrane, then $\frac{\mathrm{d}^2\varphi}{\mathrm{d}x^2} = 0$. We define electric field strength (E) within the membrane:

$$E = -\frac{\mathrm{d}\varphi}{\mathrm{d}x} = \frac{\varphi(x=0) - \varphi(x=d)}{d} \tag{S28}$$

In this equation, $\varphi(x=0)$ and $\varphi(x=d)$ are the membrane-side potentials at the membrane-anolyte interface and the membrane-catholyte interface, respectively. Therefore,

$$\frac{\mathrm{d}}{\mathrm{d}x}\left(\frac{\mathrm{d}c_i}{\mathrm{d}x}\right) = \frac{FE}{RT}\frac{\mathrm{d}c_i}{\mathrm{d}x} \tag{S29}$$

Then, we have:

$$\frac{\mathrm{d}c_i}{\mathrm{d}x} = Ae^{\frac{FE}{RT}x} \tag{S30}$$

$$c_i = A\frac{RT}{FE}e^{\frac{FE}{RT}x} + B \tag{S31}$$

A and B are constants to be determined. Then, J_i can be expressed as:

$$J_i = -D_iAe^{\frac{FE}{RT}x} + \frac{F}{RT}D_iE\left(A\frac{RT}{FE}e^{\frac{FE}{RT}x} + B\right) = \frac{F}{RT}D_iEB \tag{S32}$$

We use the boundary conditions at x=0 and x=d:

$$A\frac{RT}{FE} + B = c_i(x=0) \tag{S33}$$

$$A\frac{RT}{FE}e^{\frac{FE}{RT}d} + B = c_i(x=d) \tag{S34}$$

Herein, $c_i(x=0)$ and $c_i(x=d)$ are the membrane-side concentration of species i at the membrane-anolyte interface and membrane-catholyte interface, respectively. By adopting 1st order Taylor expansion, we get:

$$A\frac{RT}{FE}(1 + \frac{FE}{RT}d) + B = c_i(x=d) \tag{S35}$$

Then, we have:

$$B = c_i(x=0) + \frac{RT}{FEd}[c_i(x=0) - c_i(x=d)] \tag{S36}$$

$$\begin{aligned} J_i &= D_i\frac{c_i(x=0)-c_i(x=d)}{d} + \frac{FD_i}{RT}Ec_i(x=0) \\ &= D_i\frac{c_i(x=0)-c_i(x=d)}{d} + \frac{FD_i}{RTd}[\varphi(x=0) - \varphi(x=d)]c_i(x=0) \end{aligned} \tag{S37}$$

Therefore, the steady-state total flux through the membrane can be expressed as the sum of two parts. One part is linear to the concentration difference between the two

sides. The other part is linear to the potential difference between the two sides and the concentration of one side.

Donnan potential $\Delta\varphi_\mathrm{D}$, the potential difference at the membrane-electrolyte interface, follows the relation:

$$\Delta\varphi_\mathrm{D} = \varphi(x=0) - \varphi_\mathrm{a} = \frac{RT}{F}\ln\frac{c_{i,\mathrm{a}}}{c_i(x=0)} = \varphi(x=d) - \varphi_\mathrm{c} = \frac{RT}{F}\ln\frac{c_{i,\mathrm{c}}}{c_i(x=d)} \quad \text{(S38)}$$

In this equation, φ_a and φ_c are the potential of the anolyte and catholyte, respectively. $c_{i,\mathrm{a}}$ and $c_{i,\mathrm{c}}$ are the concentration of species i in the anolyte and catholyte, respectively. Therefore, the expression of J_i can be written as:

$$J_i = D_i e^{-\frac{F\Delta\varphi_\mathrm{D}}{RT}}\frac{c_{i,\mathrm{a}}-c_{i,\mathrm{c}}}{d} + \frac{FD_i}{RTd}e^{-\frac{F\Delta\varphi_\mathrm{D}}{RT}}[\varphi_\mathrm{a} - \varphi_\mathrm{c}]c_{i,\mathrm{a}} \quad \text{(S39)}$$

This equation has a similar form as:

$$J_i = k_{i,\mathrm{d}}(c_{i,\mathrm{a}} - c_{i,\mathrm{c}}) + k_{i,\mathrm{m}}[\varphi_\mathrm{a} - \varphi_\mathrm{c}]c_{i,\mathrm{a}} \quad \text{(S40)}$$

Supplementary Note 3. Approximation in equilibrium calculation

In the equilibrium calculation under CO_2, the first proton dissociation of H_2CO_3 was considered. The further proton dissociation of HCO_3^- and autoionization of H_2O were neglected. This is due to the fact the equilibrium constants of water autoionization ($K_w = 1.0\times10^{-14}$) and the second proton dissociation of H_2CO_3 ($K_{a2} = 4.7\times10^{-11}$) are far less than the equilibrium constant of first proton dissociation of H_2CO_3 ($K_{a1} = 4.5\times10^{-7}$) and the catholyte was saturated by CO_2. The concentration of HCO_3^-, CO_3^{2-} and OH^- can be expressed as:

$$[\mathrm{HCO_3^-}] = \frac{K_{a1} h p_{\mathrm{CO_2}}}{[\mathrm{H^+}]} \quad \text{(S41)}$$

$$[\mathrm{CO_3^{2-}}] = \frac{K_{a2}[\mathrm{HCO_3^-}]}{[\mathrm{H^+}]} \quad \text{(S42)}$$

$$[\mathrm{OH^-}] = \frac{K_w}{[\mathrm{H^+}]} \quad \text{(S43)}$$

Then, the $[CO_3^{2-}]/[HCO_3^-]$ and $[OH^-]/[HCO_3^-]$ ratios can be expressed as:

$$[\mathrm{CO_3^{2-}}]/[\mathrm{HCO_3^-}] = K_{a2}/[\mathrm{H^+}] \quad \text{(S44)}$$

$$[\mathrm{OH^-}]/[\mathrm{HCO_3^-}] = K_w/K_{a1} h p_{\mathrm{CO2}} \quad \text{(S45)}$$

Considering that the pH varied in the range between 2 and 6 in CO_2 atmosphere as indicated by simulation and experiment, both ratios above are much less than 1. Namely, $[CO_3^{2-}] << [HCO_3^-]$ and $[OH^-] << [HCO_3^-]$. Therefore, the shifts of the equilibrium of second proton dissociation of H_2CO_3 and autoionization of water lead to negligible change of $[HCO_3^-]$. The equilibrium between HCO_3^- and CO_2 determines the pH of electrolyte. Therefore, the shifts of the equilibrium of second proton dissociation of H_2CO_3 and autoionization of water should have negligible effect on the pH of the electrolyte.

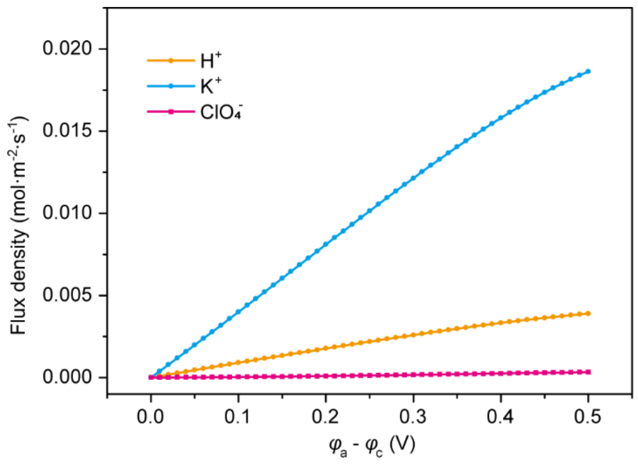

Figure S3. The comparison among the flux densities of H^+, K^+ and ClO_4^- through the Nafion 117 membrane from the PNP simulation. In the simulation, $c_{H+,a}$, $c_{H+,c}$, $c_{K+,a}$ and $c_{K+,c}$ were fixed at 0.1 M, φ_c was fixed at 0 V and φ_a was varied.

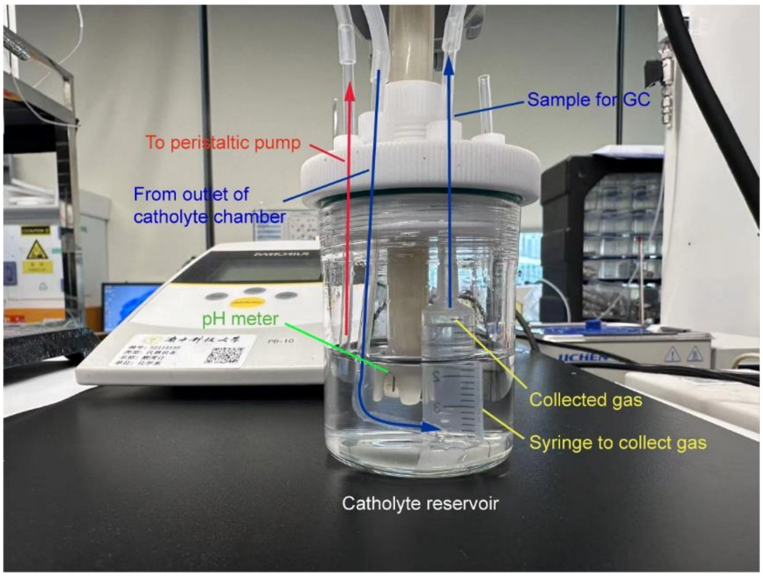

Figure S4. Photo of the catholyte reservoir with the upside-down syringe to collect the gas from the outlet of the catholyte chamber of the flow cell.

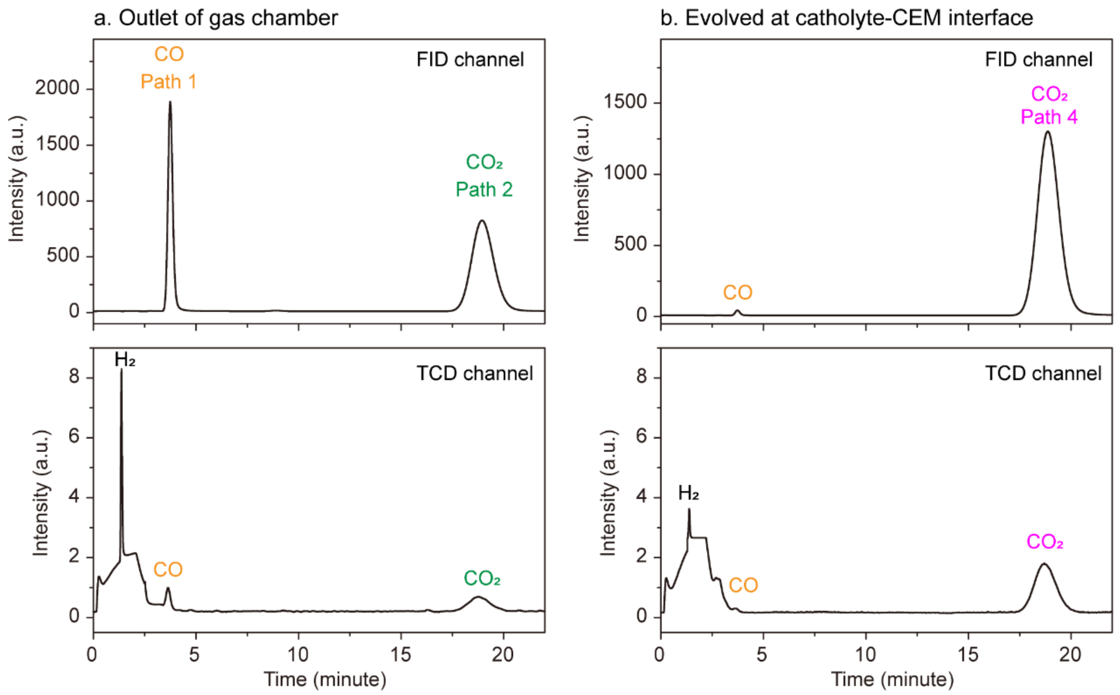

Figure S5. GC traces of the gas from the outlet of the gas chamber of the cell (a) and the gas evolved from at the catholyte-CEM interface collected from the outlet of the catholyte chamber of the cell (b). CO detected in panel (b) was also considered when calculating the FE of CO. The electrolyte was 0.05 M H_2SO_4 + 0.1 M K_2SO_4 and the GC curves were collected at 2 hours of electrolysis.

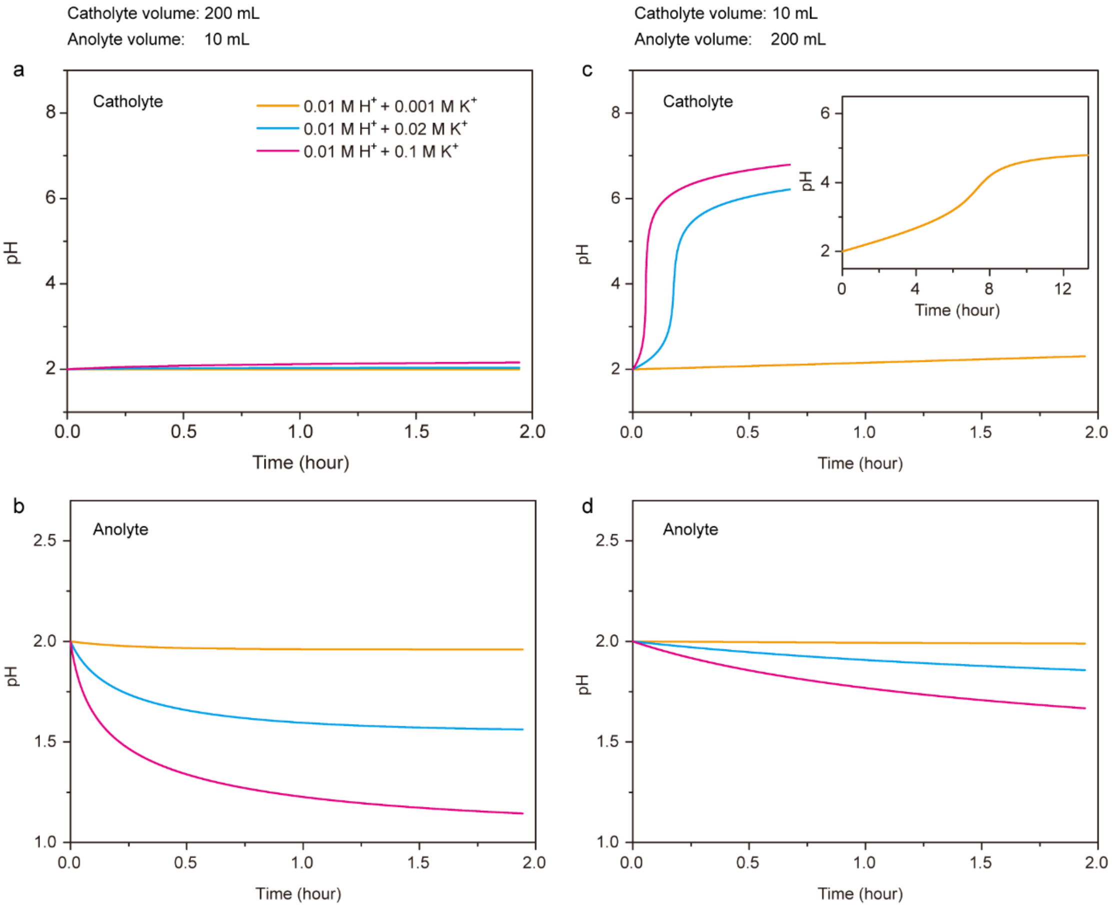

Figure S6. Simulated pH variation of the electrolytes with different volumes of the catholyte and the anolyte. (a, b) The volumes of catholyte and anolyte are 200 mL and 10 mL, respectively. (c, d) The volumes of catholyte and anolyte are 10 mL and 200 mL, respectively. The inset of (c) shows the pH of catholyte with the initial composition of 0.01 M $HClO_4$ + 0.001 M $KClO_4$ in 13 hours of electrolysis.

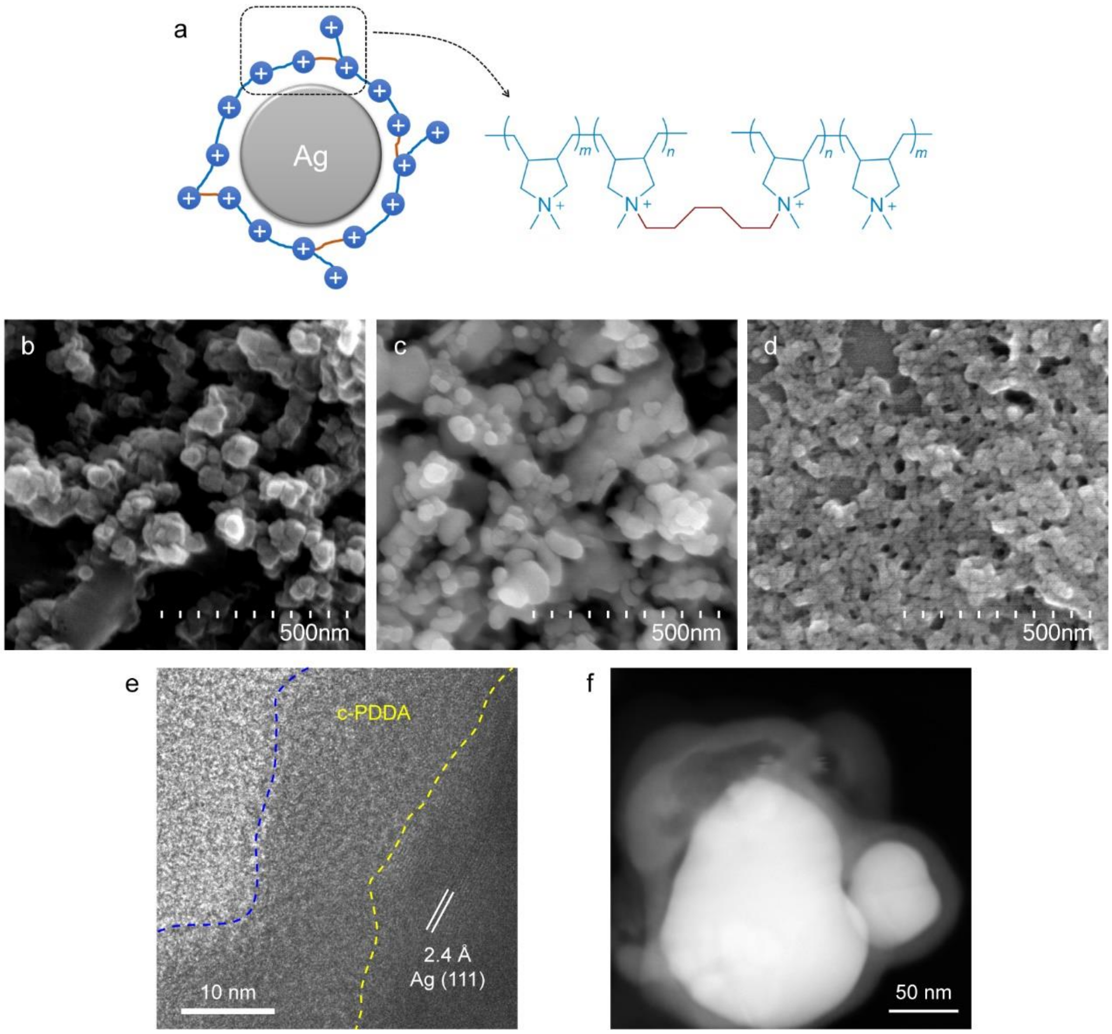

Figure S7. (a) Schematic of c-PDDA decorated Ag nanoparticle. SEM image of the micropore layer of GDE (b), Ag nanoparticles loaded on the micropore layer of GDE (c) and c-PDDA decorated Ag nanoparticles (d). HRTEM (e) and HAADF-STEM (f) images of c-PDDA decorated Ag nanoparticle.

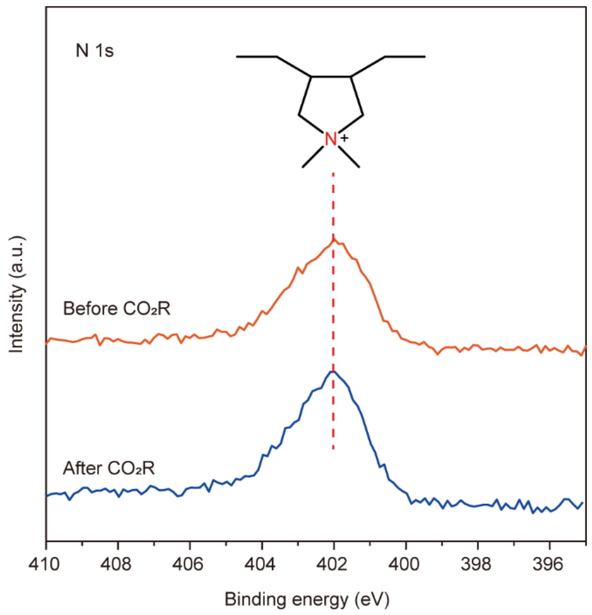

Figure S8. N 1s XPS spectra of GDE before and after CO_2R electrolysis in 0.05 M H_2SO_4.

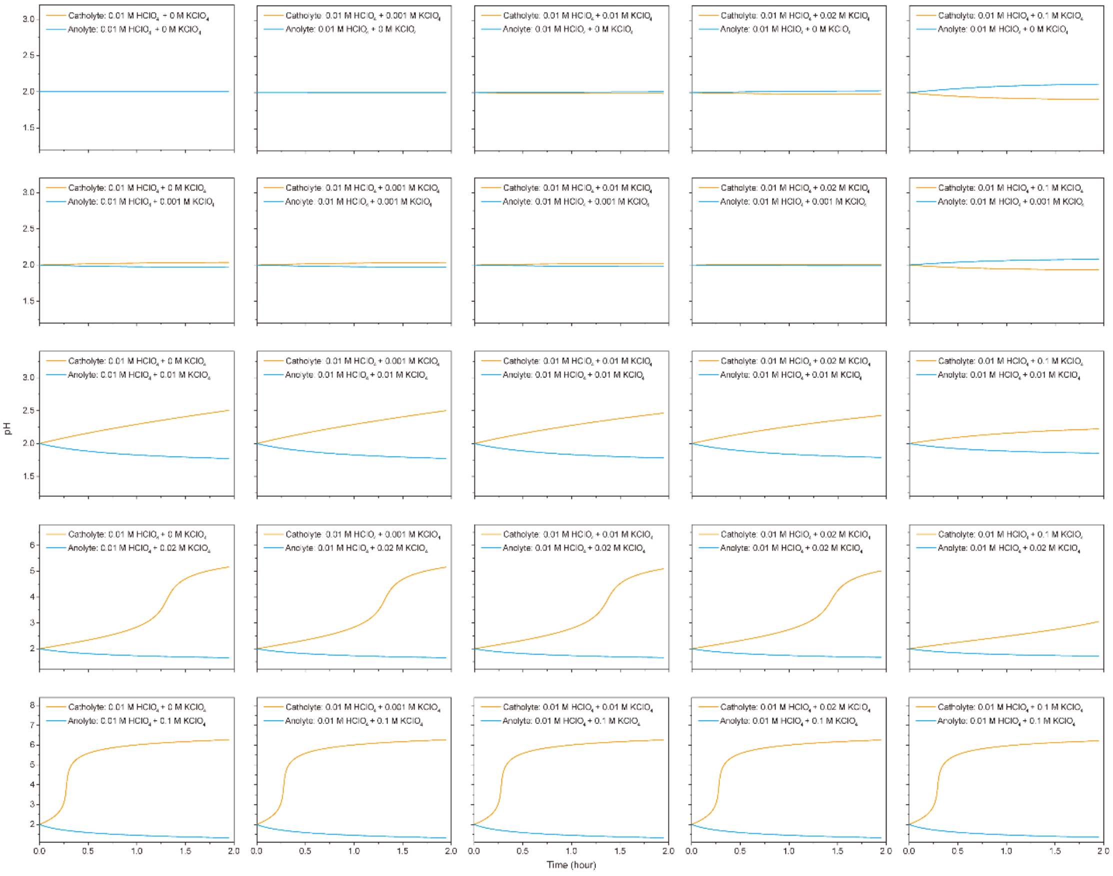

Figure S9. Simulated pH curves of the catholyte and the anolyte under CO_2 atmosphere. The initial catholyte and anolyte contained 0.01 M of $HClO_4$ and varied concentration of $KClO_4$.

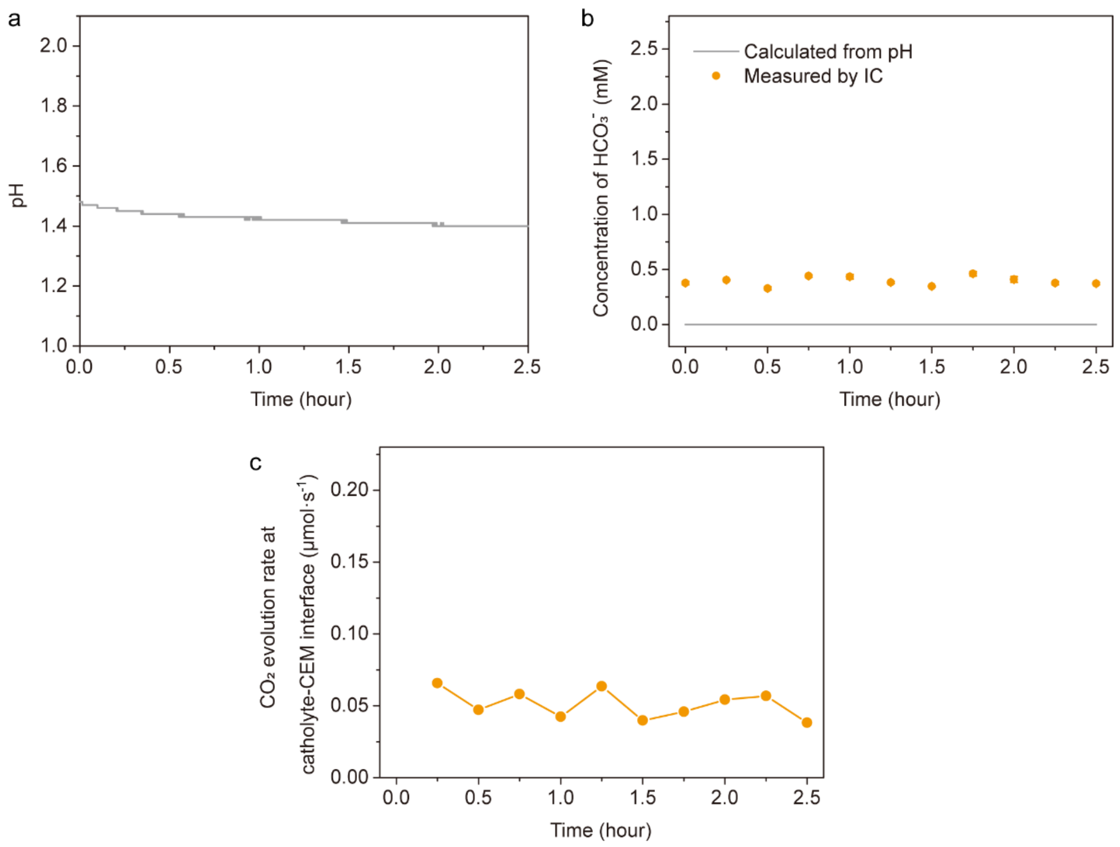

Figure S10. Concentration of HCO_3^- in the catholyte and formation rate of CO_2 at the catholyte-CEM interface. 0.05 M H_2SO_4 + 0.1 M K_2SO_4 was used as the initial catholyte and 0.05 M H_2SO_4 was used as the initial anolyte. (a) pH of the catholyte. (b) Concentration of HCO_3^- in the catholyte. The grey curve indicates the concentration of HCO_3^- calculated from the pH values and the orange spots indicate the concentration of HCO_3^- measured by IC. The error bars are the standard derivatives of three measurements. The catholyte sample was degassed before IC measurement. The HCO_3^- concentration measured by IC was higher because of the remaining CO_2 dissolved in the catholyte, similar to Figure 3a-c in the main text. (c) Formation rate of CO_2 at the catholyte-CEM interface measured by GC.

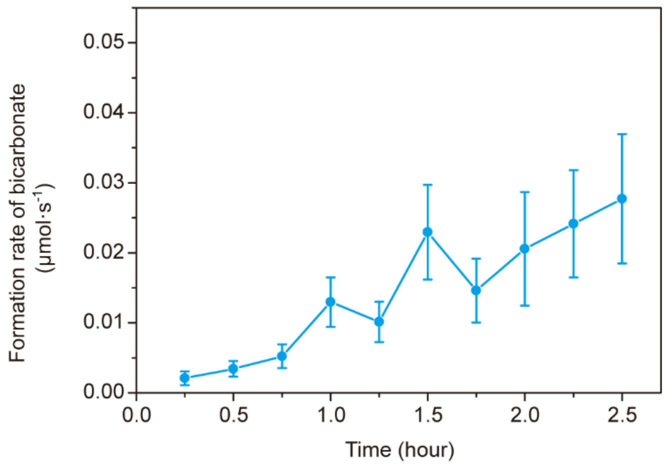

Figure S11. Formation rate of bicarbonate precipitate during CO_2R electrolysis with 0.05 M H_2SO_4 + 0.1 M K_2SO_4 as the initial catholyte and 0.05 M H_2SO_4 as the initial anolyte. The cathode GDE was rinsed with 10 mL of deionized water from the gas-chamber side every 0.25 hour, then the concentration of HCO_3^- in the rinsed water was quantified by IC. The error bars are the standard derivatives of three measurements.

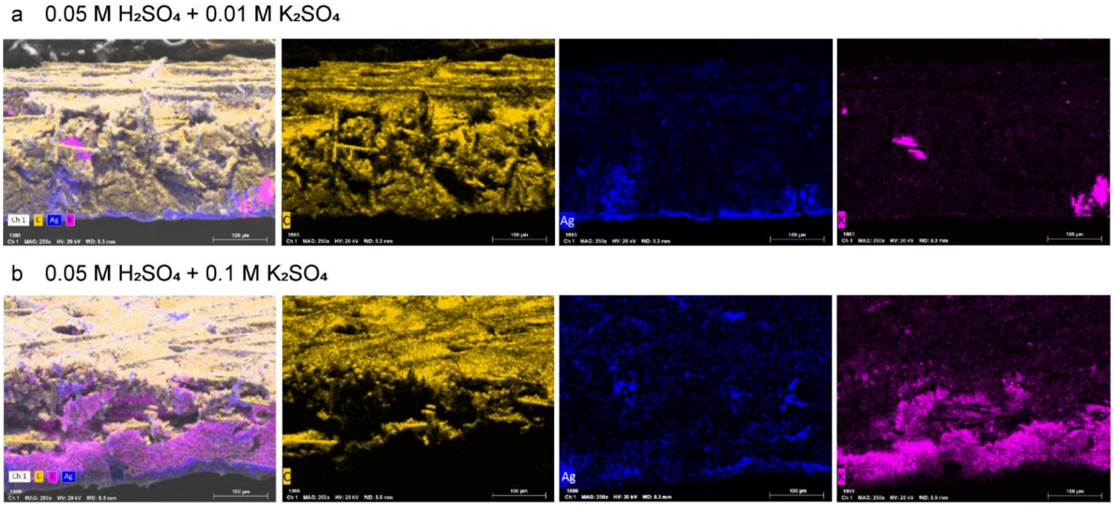

Figure S12. SEM and EDS mapping images of the cross-section of the working electrode after CO_2R electrolysis in 0.05 M H_2SO_4 + 0.01 M K_2SO_4 (a) and 0.05 M H_2SO_4 + 0.1 M K_2SO_4 (b). The images in each row from left to right are the merged image of SEM and EDS mapping, EDS mapping of C element, Ag element and K element, respectively.

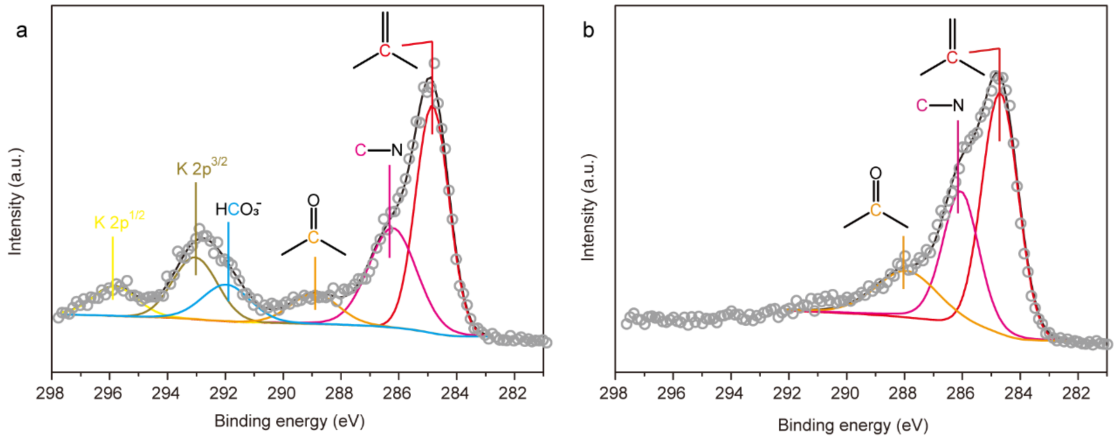

Figure S13. C 1s and K 2p XPS spectra of the GDE after CO_2R electrolysis in 0.05 M H_2SO_4 + 0.1 M K_2SO_4 (a) and in 0.05 M H_2SO_4 (b).

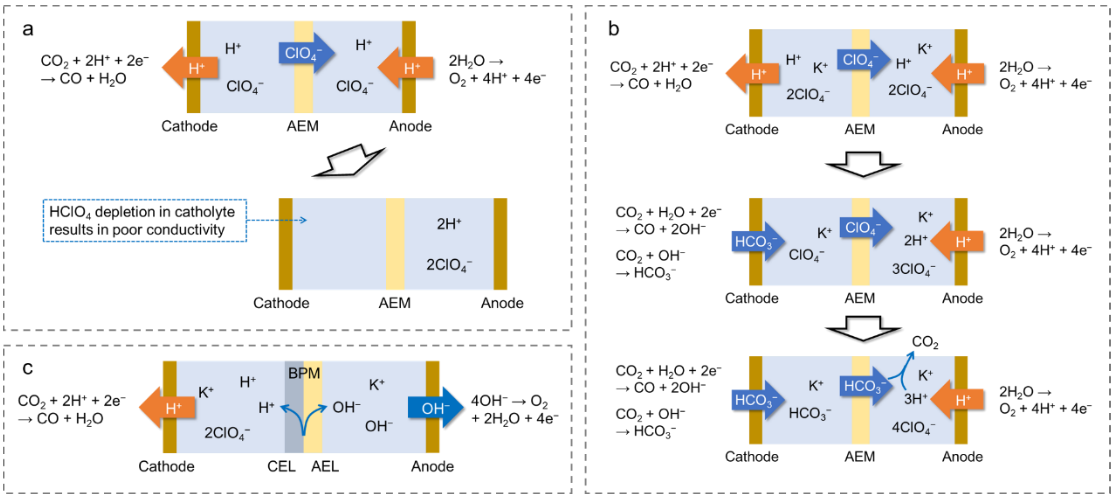

Figure S14. Schematic illustrations of electrolysis cell for acidic CO2R with AEM and BPM. (a) The AEM-based cell with the pure acid solution as the initial catholyte and anolyte. (b) The AEM-based cell with acidic solution containing alkali cations as the initial catholyte and anolyte. (c) The BPM-based cell with acidic solution containing alkali cations as the catholyte and alkaline solution as the anolyte.

Table S1. Values of parameters used in the PNP simulation.

Parameter	Description	Value	Unit	Reference
$D_{H+,sol}$	Diffusion coefficient of H^+ in solution	9.311×10^{-9}	$m^2\cdot s^{-1}$	[3]
$D_{H+,CEM}$	Diffusion coefficient of H^+ in CEM	4.6×10^{-10}	$m^2\cdot s^{-1}$	[4]
$D_{K+,sol}$	Diffusion coefficient of K^+ in solution	1.957×10^{-9}	$m^2\cdot s^{-1}$	[3]
$D_{K+,CEM}$	Diffusion coefficient of K^+ in CEM	1.1×10^{-10}	$m^2\cdot s^{-1}$	[4]
$D_{ClO4-,sol}$	Diffusion coefficient of ClO_4^- in solution	1.792×10^{-9}	$m^2\cdot s^{-1}$	[5]
$D_{ClO4-,CEM}$	Diffusion coefficient of ClO_4^- in CEM	9.0×10^{-11}	$m^2\cdot s^{-1}$	[4]
$\rho_{fix,sol}$	Fixed charge density in solution	0	$C\cdot m^{-3}$	
$\rho_{fix,CEM}$	Fixed charge density in CEM	-9.6×10^{7}	$C\cdot m^{-3}$	[2]
ε_0	Permittivity of vacuum	8.8542×10^{-12}	$F\cdot m^{-1}$	[3]
$\varepsilon_{r,sol}$	Relative permittivity of water	80.1		[3]
$\varepsilon_{r,CEM}$	Relative permittivity of CEM	30		[6]

REFERENCES

[1] K.A. Mauritz, R.B. Moore, *Chem. Rev.* **2004**, *104*, 4535-4586.

[2] V. Kumar, P. Kumar, A. Nandy, P.P. Kundu, *RSC Adv.* **2016**, *6*, 21526-21534.

[3] D. Bohra, J.H. Chaudhry, T. Burdyny, E.A. Pidko, W.A. Smith, *Energy Environ. Sci.* **2019**, *12*, 3380-3389.

[4] H. Tang, D. Aili, R.A. Tufa, M.R. Kraglund, Q. Wu, C. Pan, L.N. Cleemann, Q. Li, *Solid State Ion.* **2022**, *383*, 115984.

[5] W.M. Haynes, D.R. Lide, T.J. Bruno, CRC Handbook of Chemistry and Physics, 97th. ed., CRC Press, Boca Raton, 2016.

[6] S.A. Mareev, E. Evdochenko, M. Wessling, O.A. Kozaderova, S.I. Niftaliev, N.D. Pismenskaya, V.V. Nikonenko, *J. Membr. Sci.* **2020**, *603*, 118010.
